# Supplementary material for: Priming Human Repopulating Hematopoietic Stem and Progenitor Cells for Cas9/sgRNA Gene Targeting
Source: Mol Ther Nucleic Acids. 2018 May 3;12:89–104. doi: 10.1016/j.omtn.2018.04.017 (PMC6023838; doi:10.1016/j.omtn.2018.04.017)
Supplement: Document S1. Figures S1–S7 [file mmc1.pdf]

OMTN, Volume *12*

Supplemental Information

Priming Human Repopulating Hematopoietic Stem and Progenitor Cells for Cas9/sgRNA Gene Targeting

Carsten T. Charlesworth, Joab Camarena, M. Kyle Cromer, Sriram Vaidyanathan, Rasmus O. Bak, Jason M. Carte, Jason Potter, Daniel P. Dever, and Matthew H. Porteus

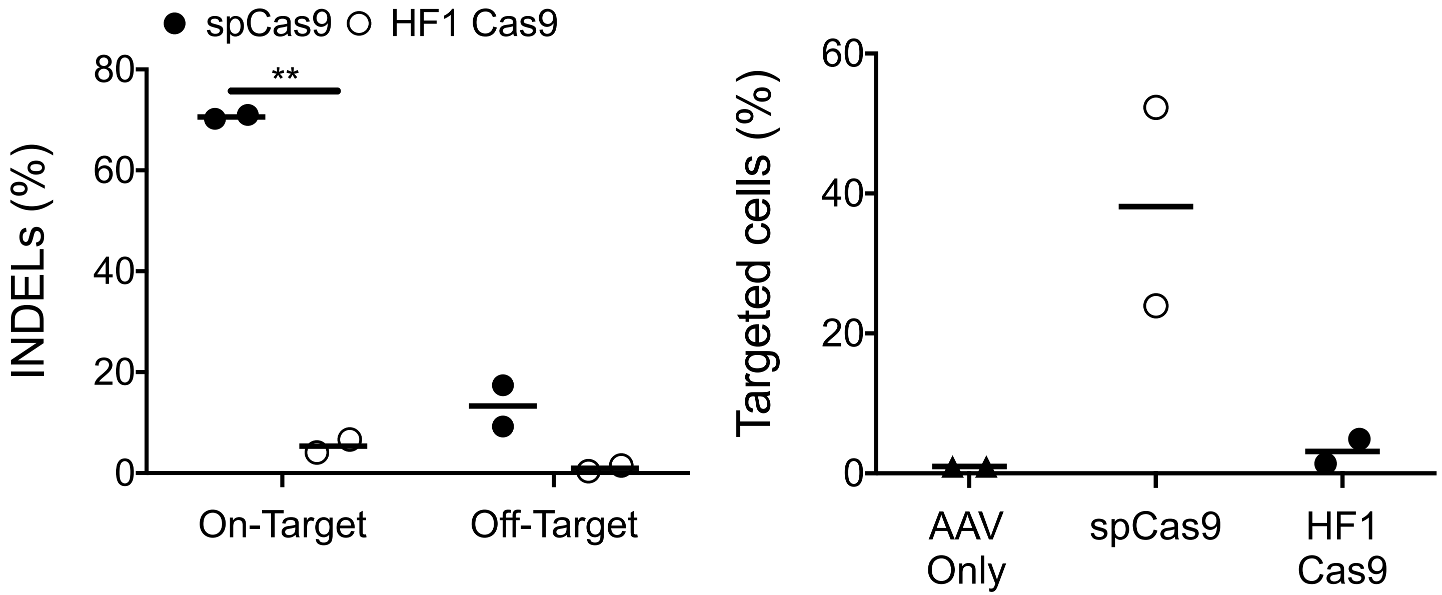

Supplemental Figure 1. CB $CD34^+$ HSPCs were edited at the *HBB* locus using either sgRNA/Cas9 only (left) or sgRNA/Cas9 plus AAV6 (right). Left, percentage of alleles that contain INDELs at the *HBB* locus and at a known off-target site for the R-02 guide when HF1 Cas9 was used to compare to the wild type spCas9 (n = number of data points). **$P < 0.05$, paired t-test. Right, AAV6-SFFV-GFP was used to deliver a homologous donor containing GFP controlled by the SFFV promoter to cells. The percentage of cells edited was measured by GFP^{high} 4 days after targeting (n = number of data points plotted). Bars represent mean.

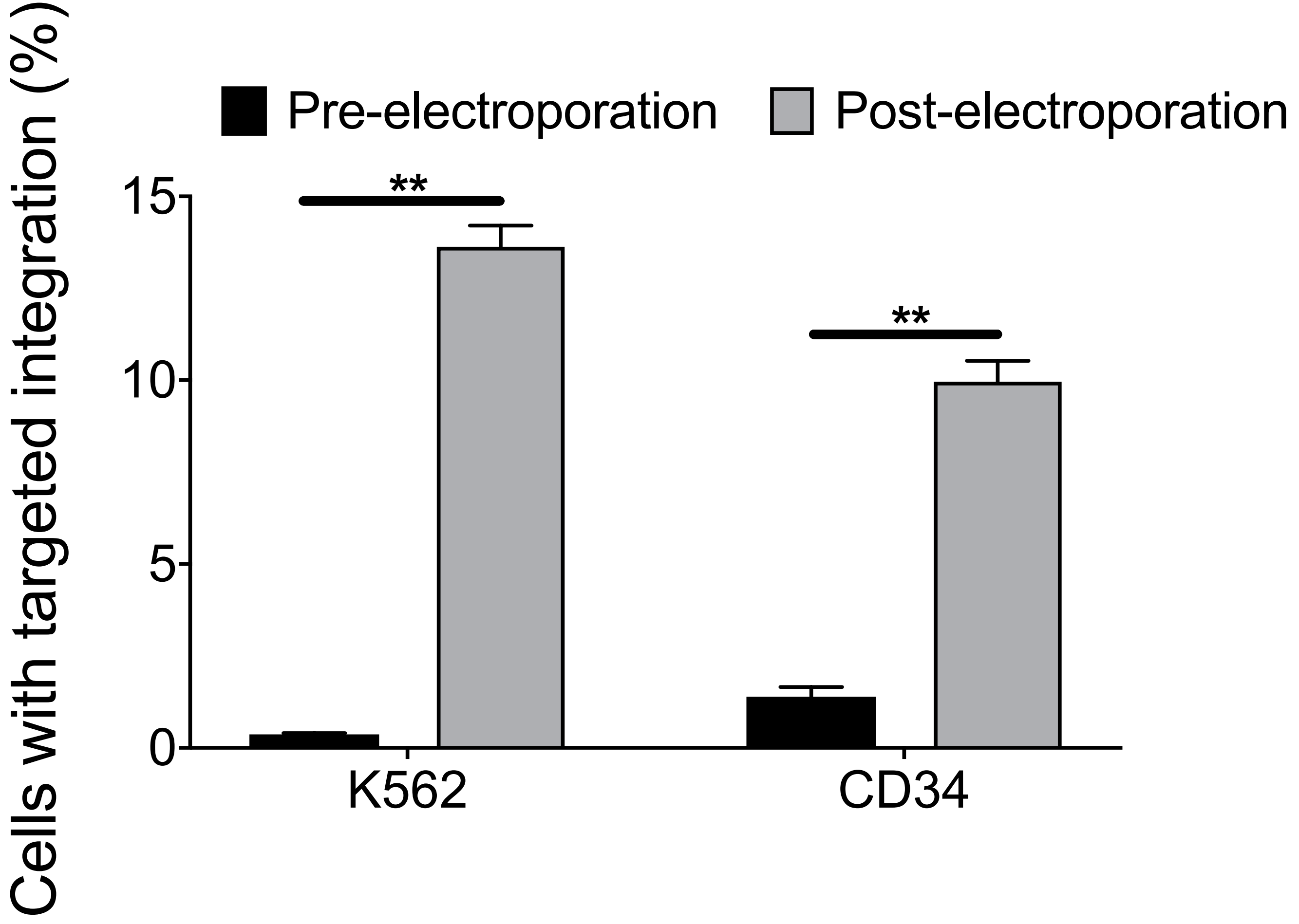

Supplemental Figure 2. Percentage of PB cells that undergo HR at the *HBB* locus when rAAV6 is added 24 hours before or directly after electroporation of cells with Cas9/sgRNA RNP at a multiplicity of infection (MOI) of $5{\times}10^4$ vg/cell (n = 3 replicates of K562 and n = 4-6 PB $CD34^+$ donors). **$P < .01$, paired t-test.

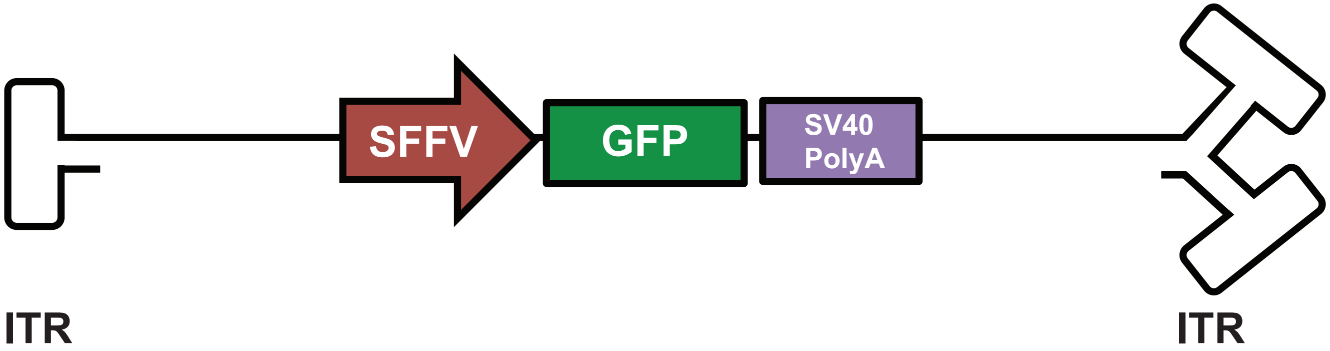

Supplemental Figure 3. Schematic showing the structure of scAAV6. ITR stands for Iinverted terminal repeat.

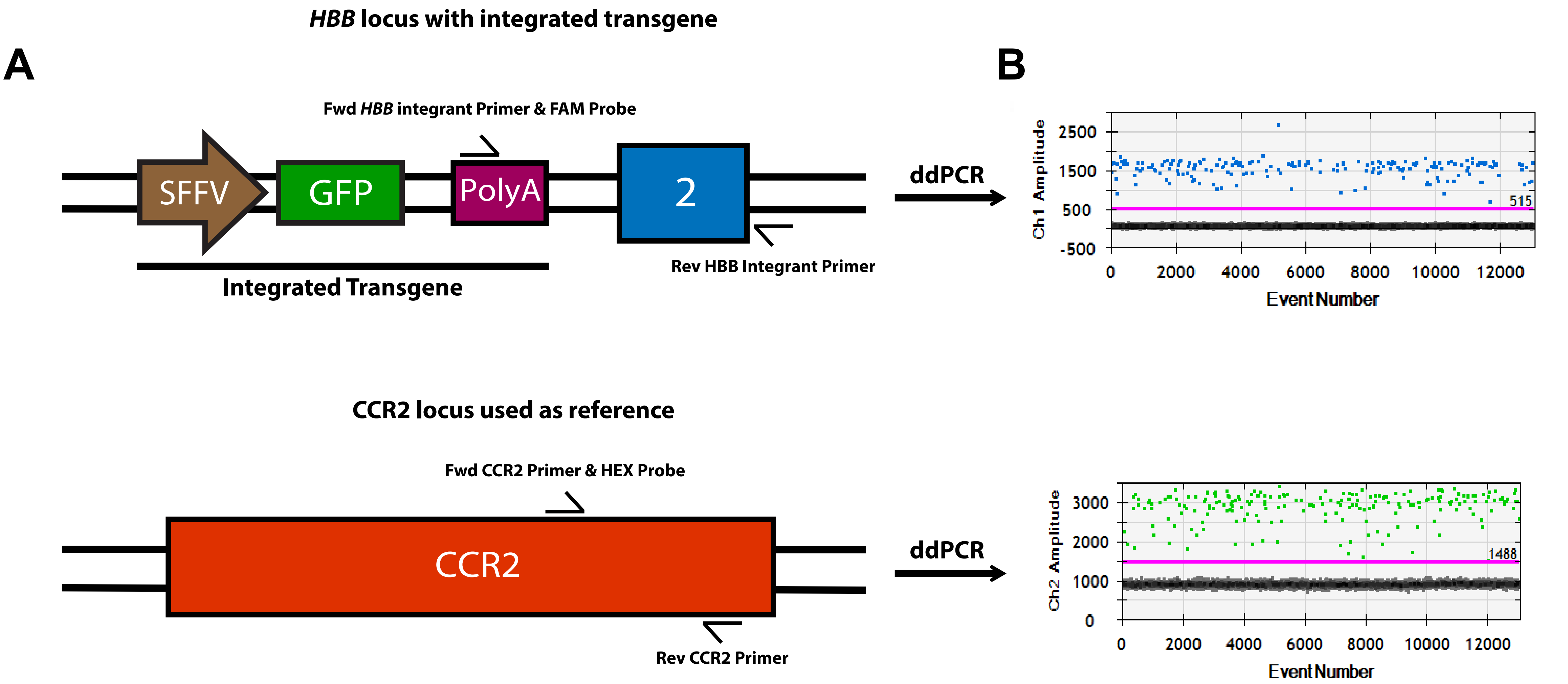

Supplemental Figure 4. Schematic demonstrating the position of probes for detecting integrated transgene donor relative to a reference gene and detection by droplet reader. **(A)** Position of primers and probes for amplifying an allele with an integrated transgene at the HBB locus using an in-out PCR (one primer binding to integrated insert with FAM probe 3 bp upstream and other primer biinding to *HBB* locus outside of the homology arm) and primers for amplifying the CCR2 locus to serve as a reference for the number of alleles edited. **(B)** Representative ddPCR readout demonstrating droplets that are positive for a transgene as detected by FAM fluorescence compared to areporter gene as detected by HEX fluorescence.

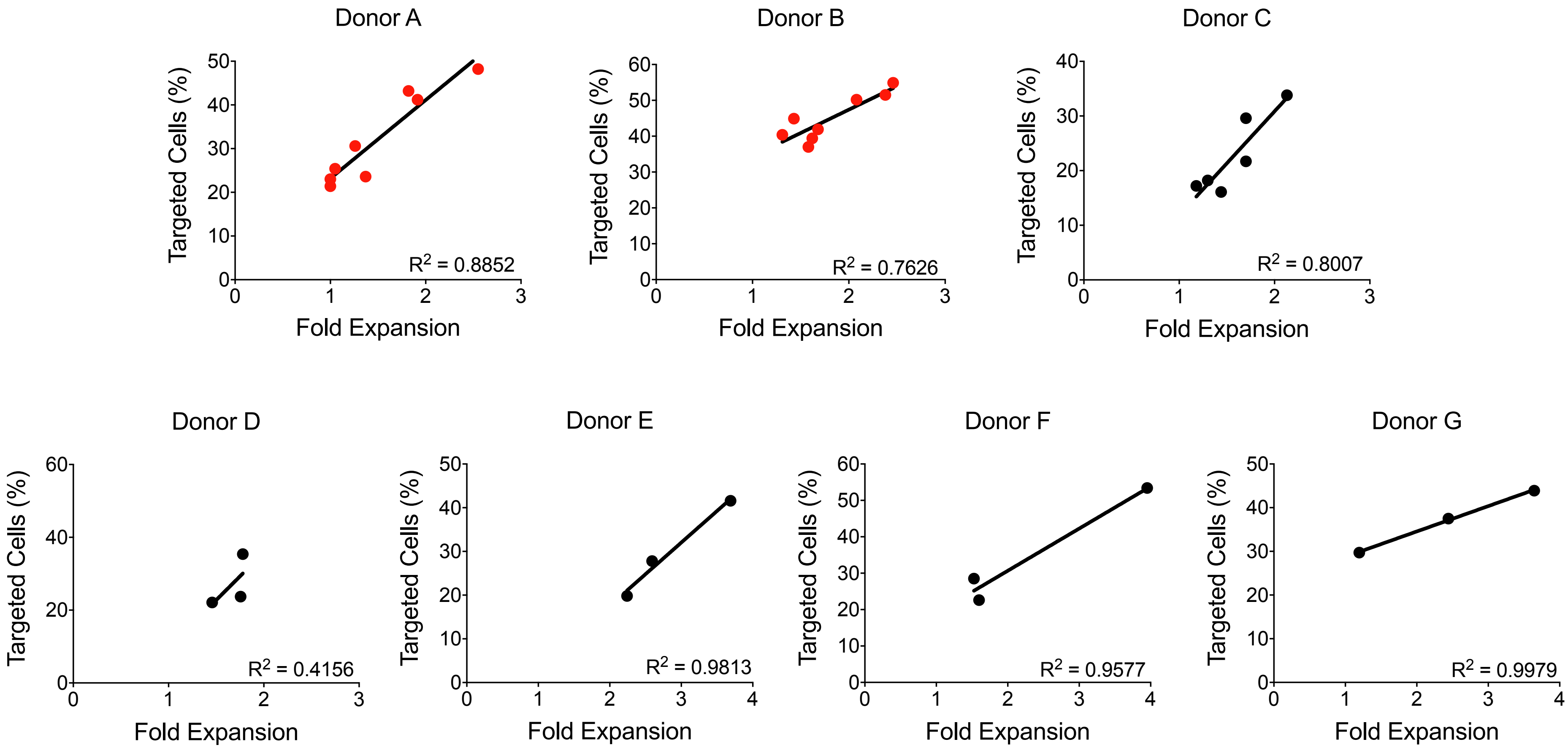

Supplemental Figure 5. Individual donors were plated at different cell densities or for different amounts of times and then targeted at the *HBB* locus. The fold expansion of cells is plotted against the % of cells in the population that underwent HR and a linear regression plotted for each donor. Red dots represent a mobilized peripheral blood donor source and black dots represent cord blood donor source.

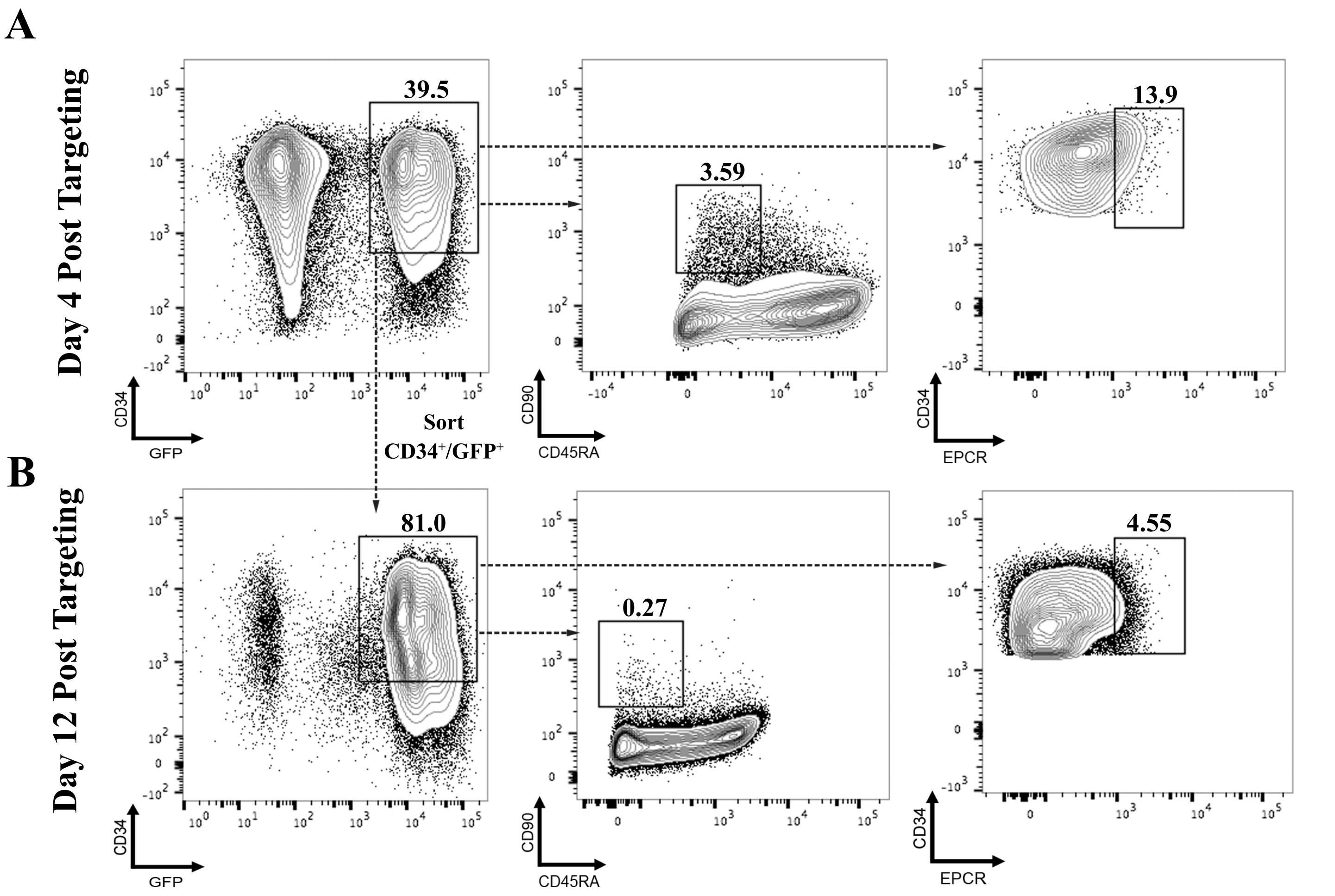

Supplemental Figure 6. Expanding CB *HBB*-targeted HSPCs for 12 days post-targeting reduces the percentage of immunophenotypic repopulating HSPCs. **A)** *HBB*-targeted HSPCs were analyzed day 4 post-targeting for $CD34^+/GFP^+$ expression (left), CD90/CD45RA expression (middle) and CD201 (EPCR) expression (right). $CD34^+/CD45RA^-/CD90^+/GFP^+$ cells indicate targeting efficiencies in repopulating HSCs. EPCR high expression (right) is indicative of HSPCs with long-term population when cultured. **B)** FACS plot showing the percentage of immunophenotypic repopulating HSPCs in *HBB*-targeted cells after 8 days of expansion at low densities. Data show the presence but a reduction in the percentage of HSPCs expressing CD90 (middle) and EPCR (right).

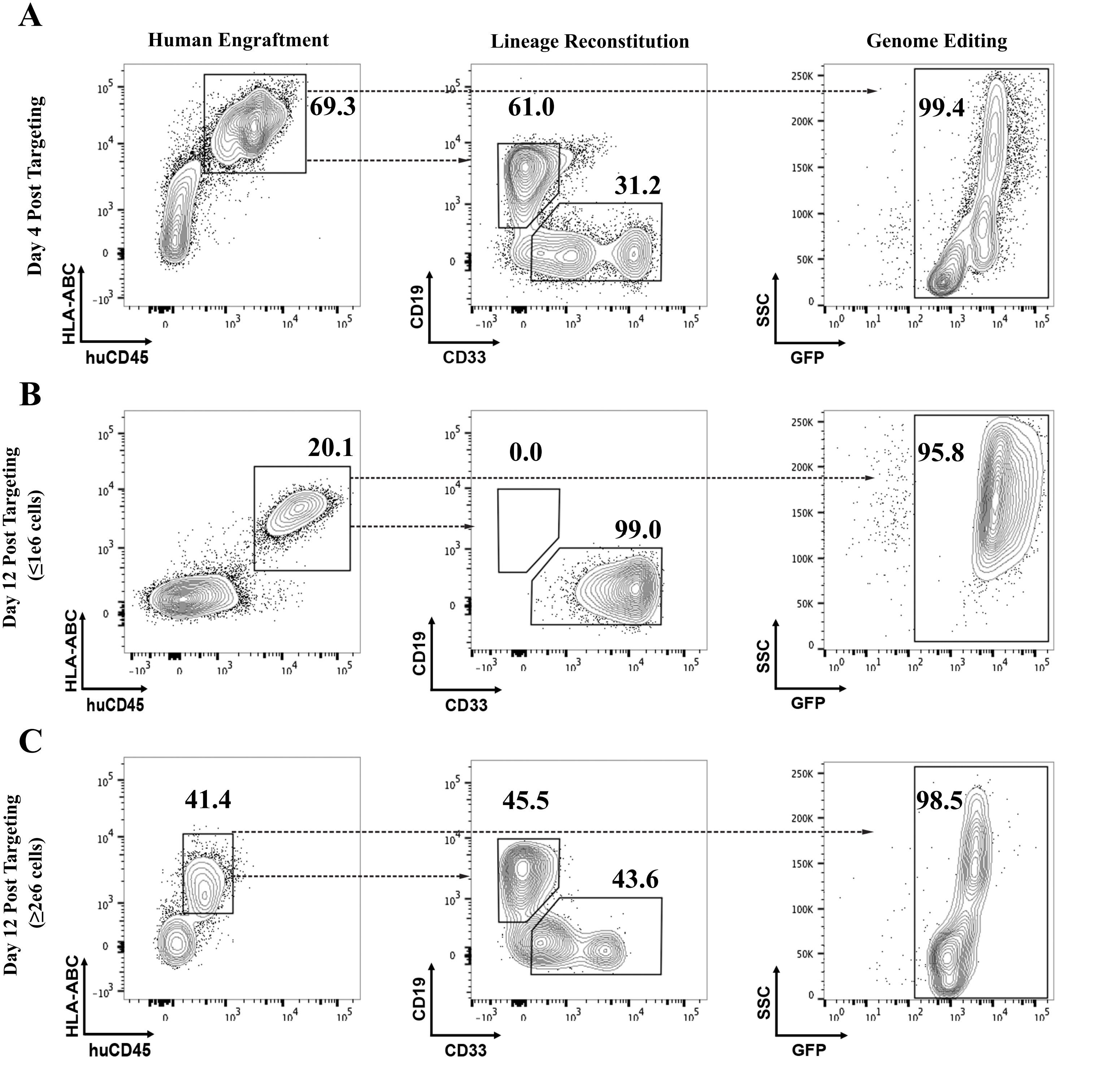

Supplemental Figure 7. Representative FACS plots from **Figure 6C-E** showing human chimerism of CB *HBB*-targeted HSPCs in NSG bone marrow. **A)** 1×10^6 $CD34^+/GFP^+$ *HBB*-targeted HSPCs day 4-post targeting were injected into the right femur of a NSG mouse. 16 weeks post transplant, the bone marrow was analyzed for human cells and further analyzed for bi-lineage reconstitution and genome editing frequencies via GFP analysis. Data shows robust human engraftment in mouse femur (left) with bi-lineage reconstitution (right) of Cas9/sgRNA modified cells (right). **B)** On day 4 post-targeting, $CD34^+/GFP^+$ HBB-targeted HSPCs were expanded for an additional 8 days and then 1×10^6 CD34+/GFP+ cells were sorted again and finally injected into the right femur of a NSG mouse. Human cells were analyzed as above. Data shows robust engraftment of human cells, but with only myeloid reconstitution. **C)** On day 4 post-targeting, 5×10^4 $CD34^+/GFP^+$ *HBB*-targeted HSPCs were sorted and expanded 57.5 fold for an additional 8 days at low densities and then injected in the right femur of a NSG mouse. Human cell chimerism, lineage reconstitution and genome editing was analyzed at 16 weeks post-targeting. Data show robust engraftment compared to Day 4 equivalent (5×10^4 ; **Figure 6E**) as well as multi-lineage reconstitution (middle) of a pure genome edited population (right).
