# Supplementary material for: Priming Human Repopulating Hematopoietic Stem and Progenitor Cells for Cas9/sgRNA Gene Targeting
Source: Mol Ther Nucleic Acids. 2018 May 3;12:89–104. doi: 10.1016/j.omtn.2018.04.017 (PMC6023838; doi:10.1016/j.omtn.2018.04.017)
Supplement: Document S2. Article plus Supplemental Information [file mmc2.pdf]

Molecular Therapy
Nucleic Acids
Original Article

Priming Human Repopulating Hematopoietic Stem and Progenitor Cells for Cas9/sgRNA Gene Targeting

Carsten T. Charlesworth,[1,3] Joab Camarena,[1,3] M. Kyle Cromer,[1] Sriram Vaidyanathan,[1] Rasmus O. Bak,[1] Jason M. Carte,[2] Jason Potter,[2] Daniel P. Dever,[1,4] and Matthew H. Porteus[1,4]

[1]Department of Pediatrics, Stanford University, Stanford, CA 94305, USA; [2]Thermo Fisher Scientific, 5781 Van Allen Way, Carlsbad, CA 92008, USA

Engineered nuclease-mediated gene targeting through homologous recombination (HR) in hematopoietic stem and progenitor cells (HSPCs) has the potential to treat a variety of genetic hematologic and immunologic disorders. Here, we identify critical parameters to reproducibly achieve high frequencies of RNA-guided (single-guide RNA [sgRNA]; CRISPR)-Cas9 nuclease (Cas9/sgRNA) and rAAV6-mediated HR at the β-globin (*HBB*) locus in HSPCs. We identified that by transducing HSPCs with rAAV6 post-electroporation, there was a greater than 2-fold electroporation-aided transduction (EAT) of rAAV6 endocytosis with roughly 70% of the cell population having undergone transduction within 2 hr. When HSPCs are cultured at low densities (1 × 10^5 cells/mL) prior to *HBB* targeting, HSPC expansion rates are significantly positively correlated with HR frequencies *in vitro* as well as in repopulating cells in immunodeficient NSG mice *in vivo*. We also show that culturing fluorescence-activated cell sorting (FACS)-enriched *HBB*-targeted HSPCs at low cell densities in the presence of the small molecules, UM171 and SR1, stimulates the expansion of gene-edited HSPCs as measured by higher engraftment levels in immunodeficient mice. This work serves not only as an optimized protocol for genome editing HSPCs at the *HBB* locus for the treatment of β-hemoglobinopathies but also as a foundation for editing HSPCs at other loci for both basic and translational research.

INTRODUCTION

β-hemoglobinopathies are a common group of genetic blood disorders, encompassing sickle cell disease (SCD) and β-thalassemia, which affect millions of people worldwide.[1] SCD is caused by a point mutation at codon 6 in the β-globin gene (*HBB*), resulting in a glutamate to valine substitution.[2] In the deoxygenated state, hemoglobin containing mutant β-globin polymerizes. This polymerization causes red blood cells (RBCs) to adopt a sickle shape, which leads to reduced RBC lifespan and vaso-occlusion.[3] Vaso-occlusions can lead to strokes, bone pain, kidney damage, and acute chest syndrome, impairing both quality of life and survival.[1] On the other hand, β-thalassemia is caused by multiple mutations in the *HBB* locus and is characterized by insufficient production of β-globin protein. Consequently, unpaired α-globin chains within RBC precursors initiate premature RBC death and severe anemia.[2] Currently, the only curative treatment for the β-hemoglobinopathies is allogeneic hematopoietic stem cell transplantation (allo-HSCT), a process whereby the patient receives long-term hematopoietic stem cells (LT-HSCs) with at least one non-disease causing allele from a related or non-related donor (after myeloablative conditioning to clear the stem cell niche), ultimately replacing the hematopoietic system of the patient.[1] However, allo-HSCT has important limitations, including limited availability of immunologically matched donors, increased susceptibility to infections post-allo-HSCT, and the risk of graft-versus-host disease.[2] Recent clinical studies using lentiviral gene delivery have demonstrated the potential for gene replacement therapy in LT-HSCs to improve clinical outcomes in patients suffering from β-hemoglobinopathies; however, the risk of insertional mutagenesis and transgene silencing remains a long-term safety concern.[4] Recent advances in genome editing utilizing the Cas9/single-guide RNA (sgRNA) system to mediate precise homologous recombination (HR) in hematopoietic stem and progenitor cells (HSPCs) to functionally correct β-hemoglobinopathy mutations may result in improved treatment alternatives for the still unmet medical needs of patients.[5,6]

The Cas9/sgRNA gene editing system is adapted from the CRISPR bacterial adaptive immunity system[7] that is comprised of a Cas9 nuclease (derived from *Streptococcus pyogenes* in this case) that complexes with a chimeric sgRNA, creating a ribonucleoprotein (RNP) complex. The RNP creates a DNA double-strand break (DSB) at the target site. A DSB induced by the Cas9/sgRNA system can be repaired by two repair pathways: non-homologous end-joining (NHEJ) or HR. In the NHEJ pathway, the DSB ends are re-ligated, which can result in insertions and deletions (indels) of DNA at the site of the

Received 11 March 2018; accepted 28 April 2018;
https://doi.org/10.1016/j.omtn.2018.04.017.

[3]These authors contributed equally to this work.

[4]These authors contributed equally to this work.

Correspondence: Matthew H. Porteus, Department of Pediatrics, Stanford University, Stanford, CA 94305, USA.
E-mail: mporteus@stanford.edu
Correspondence: Daniel P. Dever, Department of Pediatrics, Stanford University, Stanford, CA 94305, USA.
E-mail: ddever1@stanford.edu

This is an open access article under the CC BY license (http://creativecommons.org/licenses/by/4.0/).

DSB. By contrast, when a cell repairs a DSB through HR, it uses donor DNA homologous to the site of the DSB as a template for precise repair.[8]

The HR pathway can be co-opted to introduce a desired stretch of DNA at a specific locus when a donor template homologous to the site of the DSB is delivered into a cell by an integration-defective lentivirus (IDLV) or a recombinant adeno-associated virus serotype 6 (rAAV6).[9–11] A similar genomic outcome can be achieved by delivering the donor as a single-stranded oligonucleotide (ssODN) using a mechanistically distinct form of HR called single-stranded template repair (SSTR).[12] We and others have recently achieved precise gene correction in HSPCs by creating a DSB using the Cas9/sgRNA system followed by delivery of a donor for repair using rAAV6.[5,9,13–15] Furthermore, our group has shown that HSPCs that have undergone HR by the Cas9/sgRNA/rAAV6 platform can be identified two to four days post-targeting by a significant shift in reporter gene expression (Reporter^{high}), which allows for rapid detection and selection of edited HSPCs.[5,16–18] Thus, the use of the Cas9/sgRNA system together with rAAV6 vectors has substantial potential as a platform to edit HSPCs *ex vivo* for both basic and translational research.[5]

Here, we present a Cas9/sgRNA-rAAV6 genome-editing platform for HR in HSPCs, specifically at the *HBB* locus for the treatment of the β-hemoglobinopathies. Notably, we established that our Cas9/sgRNA system stimulates high frequencies of editing at the *HBB* locus in LT-HSCs, identified a process we have defined as electroporation-aided transduction (EAT) of rAAV6 that consistently increases rates of HR in HSPCs, and characterized a range of promoters for enrichment of targeted cells. Furthermore, we identified that low-density culture conditions drives higher frequencies of HR and determined that *ex vivo* culturing using low-density conditions supplemented with UM171/SR1 supports expansion of targeted LT-HSCs.

RESULTS

Optimizing the Delivery of *HBB* Cas9/sgRNA RNP into LT-Repopulating HSCs

Prior work demonstrated that the Cas9/sgRNA system delivered as a RNP complex by electroporation is the most effective method for creating DSBs and stimulating HR in HSPCs.[5,6,19,20] We first sought to optimize the delivery of the Cas9/sgRNA RNP complex to maximize the number of on-target DSBs made in HSPCs while minimizing cell death and off-target effects. Focusing on the application of genome editing to treat β-hemoglobinopathies, we optimized the system using a previously described guide RNA, R-02,[5,6,21] which targets the first exon of the *HBB* gene (Figure 1A).

It has previously been shown that 2′-*O*-methyl 3′phosphorothioate (MS) modifications to the three terminal nucleotides at the 5′ and 3′ ends of the sgRNA significantly increase the ability of the Cas9/sgRNA system to induce DSBs in HSPCs when Cas9 is delivered as mRNA.[10] Furthermore, previous work with zinc finger nucleases delivered to HSPCs found that a 24-hr incubation at 30°C resulted in significantly more indels compared to incubation of cells at 37°C.[22] Based on this previous work, we determined whether *HBB* MS-modified sgRNAs were more effective compared to unmodified guides at generating DSBs in HSPCs when delivering the Cas9/sgRNA system as an RNP, which has been previously shown in primary human T cells.[10] Introducing the Cas9/sgRNA RNP by electroporation into peripheral blood mobilized CD34+ cells (PB) and harvesting cells four days later, we found a significant increase in the amount of indels generated at the *HBB* locus when the Cas9/sgRNA system was delivered as an RNP with MS-modified guides versus unmodified guides at both 30°C ($p = 0.014$) and 37°C ($p = 0.004$; Figure 1B). However, no statistically significant difference between modified or unmodified guides was found in the amount of indels made at a known off-target site.[21] There was also no significant difference found in the amount of indels made when cells were cultured at 30°C versus 37°C for 24 hr for either the MS or unmodified sgRNA (Figure 1B), distinguishing Cas9/sgRNA editing from zinc finger nuclease (ZFN)-based editing.

Having found that using MS-modified guides with cells cultured at 37°C was the most effective method for generating indels in CD34^{+} HSPCs derived from cord blood (CB), we next optimized the amount of Cas9/sgRNA RNP electroporated into cells. Following electroporation of HSPCs with a range of concentrations of Cas9 RNP, we analyzed indel frequencies at both the on-target locus and the known off-target site.[21] Indel frequencies were significantly higher at the on-target locus when the RNP was introduced at a concentration of 150–600 μg/mL compared to 75 μg/mL ($p = 0.02$). At 600 μg/mL, we found a significant decrease in cell viability while achieving no significant increase in indel formation rates (Figure 1C). Therefore, we determined that Cas9 RNP delivered at 150–300 μg/mL provides the best combination of indel frequency and cell viability.

Having optimized the concentration of Cas9 RNP, we attempted to reduce the amount of off-target breaks generated by the Cas9/sgRNA system through the use of a published high-fidelity Cas9 (HF-1).[23] Whereas we found that there were significantly less indels made at analyzed off-target sites by HF-1 in CB CD34+ cells, there was also a concurrent loss in HF-1's ability to generate clinically relevant levels of indels or stimulate gene targeting in HSPCs at the *HBB* locus (Figure S1). These data are in line with a previous publication by DeWitt et al.[6] that showed a reduction in on-target editing when using the HF-1 Cas9 with the R-02 *HBB* sgRNA.

We next compared the frequency of indels made in LT-HSCs compared to short-lived progenitors within the CD34^{+} population. The current best functional measurement of human LT-HSCs is serial transplantation in Nod scid gamma (NSG) mice.[24] Using this model, we found that there was no significant drop in indel frequencies after serial transplants into secondary recipients (58% versus 49.6%; Figure 1D). In addition, all edited CB CD34+ cells demonstrated high engraftment capacity in primary and secondary NSG mice recipients at 16 weeks after transplant (in secondary transplants, cells would have been in mice for a total of 32 weeks; Figure 1E). These results indicate that there is no significant difference in the percentage of

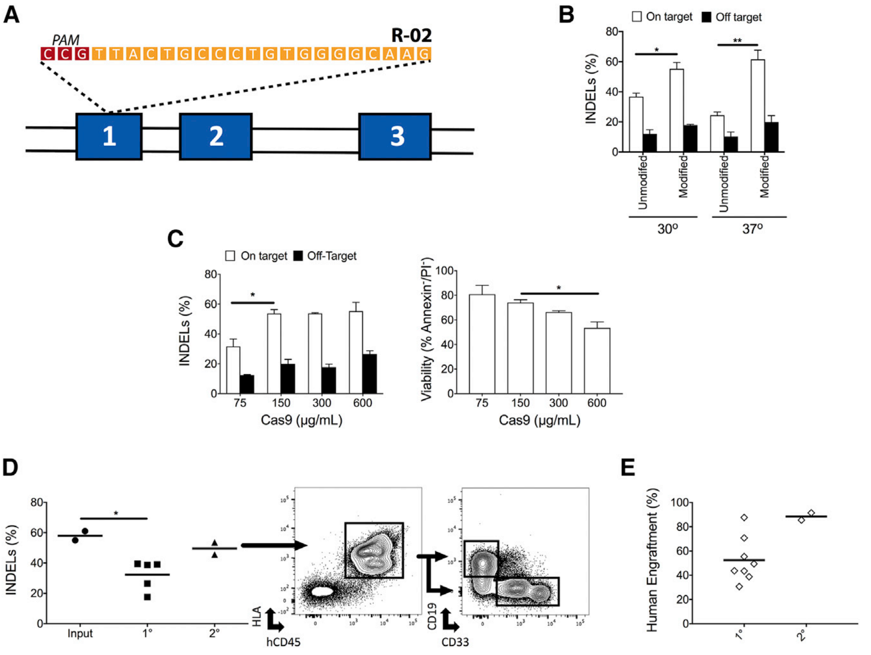

Figure 1. Electroporation of HBB-RNPs to Generate High Frequencies of Indels in Repopulating LT-HSCs

(A) Schematic representation of the genomic site at the *HBB* locus where the R-02 sgRNA binds and where Cas9 RNP generates a DSB. (B) Percent of alleles that have an indel when a MS-modified sgRNA is used compared to an unmodified sgRNA and when PB CD34+ cells are cultured at 30°C for 24 hr versus 37°C. (n = 4 for on-target and n = 3 for off-target). *p < 0.05; **p < 0.01; paired t test. (C) The left panel shows the percent of alleles containing indels at the *HBB* locus and at a known off-target site when the Cas9/sgRNA system is introduced at different concentrations per mL of nucleofection buffer. The right panel shows the viability of CB CD34+ cells one day post-electroporation with the Cas9/sgRNA RNP at different concentrations during electroporation as measured by annexin V and propidium iodide (P.I.) staining. (n = 3, except n = 2 for 300 μg/mL). *p < 0.05; unpaired t test and paired t test. (D) The left panel shows the percentage of alleles with indels in human cells before transplantation (input) and after primary (1°) and secondary (2°) transplantation into NSG mice. To the right are representative FACS plots of the gating for human CB CD34+ cells and gating for myeloid cells ($CD33^+$) and B cells ($CD19^+$) after 2° transplantation into NSG mice showing engraftment of human cells and bi-lineage reconstitution by transplanted LT-HSCs. (n = 2 different donors for input and n = number of mice analyzed for 1° and 2°). *p < 0.05; unpaired t test. (E) Frequency of engraftment of CB $CD34^+$ HSPCs post-indel formation with Cas9/sgRNA RNP in 1° and 2° transplantation into NSG mice. All values are means, and error bars represent SEM.

indels made in LT-HSCs versus short- and long-lived progenitors at the *HBB* locus using RNP delivery by electroporation.

EAT of rAAV6 in $CD34^+$ HSPCs

After optimizing the Cas9/sgRNA nuclease platform for editing *HBB* in LT-HSCs, we next investigated the optimal rAAV6 transduction parameters to best stimulate HR in HSPCs. The percentage of cells that had undergone targeted integration by HR was measured by the frequency of GFP^{high} cells as previously described.[5] We first determined the optimal time to deliver the rAAV6 donor to cells. We hypothesized that the optimal time to deliver the rAAV6 donor would be prior to electroporation of the Cas9/sgRNA system, as this would allow the rAAV6 donor to be present in the nucleus by the time that Cas9 was introduced by electroporation. This hypothesis would be consistent with a study discovering that the optimal rAAV6 delivery time relative to ZFN mRNA electroporation was 16 hr prior to electroporation.[13] Surprisingly, when we added our rAAV6 for HR donor 24 hr prior to delivery of the nuclease, in both K562 cells and PB HSPCs, we found that editing frequencies were significantly lower than when rAAV6 was added immediately after electroporation (0.36% versus 14% in K562s and 2% versus 10% in HSPCs; Figure S2). Consistent with our results, recent work has also demonstrated that the optimal time to add AAV6 to stimulate HR is after electroporation of cells.[5,14,25]

We hypothesized that the increased frequencies in targeting we observed when AAV6 was added after electroporation was due to electroporation increasing transduction of cells by AAV6. To assess this, we compared the effect of electroporation on transduction rates in CB HSPCs using a GFP-encoding self-complementary AAV6 (scAAV6-SFFV-GFP) vector with no homology arms (Figure S3). We used scAAV6 for experiments where we were analyzing AAV6 transduction into cells because self-complementary AAVs are better expressed in transduced cells than single-stranded AAVs.[26] Thus, scAAV6-SFFV-GFP is a more optimal vector to assess AAV6 tropism. Compared to non-electroporated cells, we found that electroporation yielded a greater than 2-fold increase in the overall frequency of scAAV6 transduction (Figure 2A). Notably, relative to the 48-hr time point, approximately 70% of the cells were transduced by the 2-hr time point when cells were electroporated compared to only 27% when cells were not electroporated, indicating that electroporation not only increased the overall amount of transduction by scAAV6 but also the rate at which cells are transduced; these results are consistent with the recent findings of others.[14,25] Because this effect appeared consistent across multiple cell types and with both ssAAV6 and scAAV6, we will hereafter refer to this reproducible effect as EAT.

By electroporating K562 cells and then transducing with scAAV6 at various time points post-electroporation, we found that the EAT effect was short lived (Figure 2B). Whereas electroporation increased transduction 2.5-fold when scAAV6 was added immediately after electroporation, the EAT effect was significantly decreased after 30 min, diminished by 34% after 1 hr, and completely dissipated

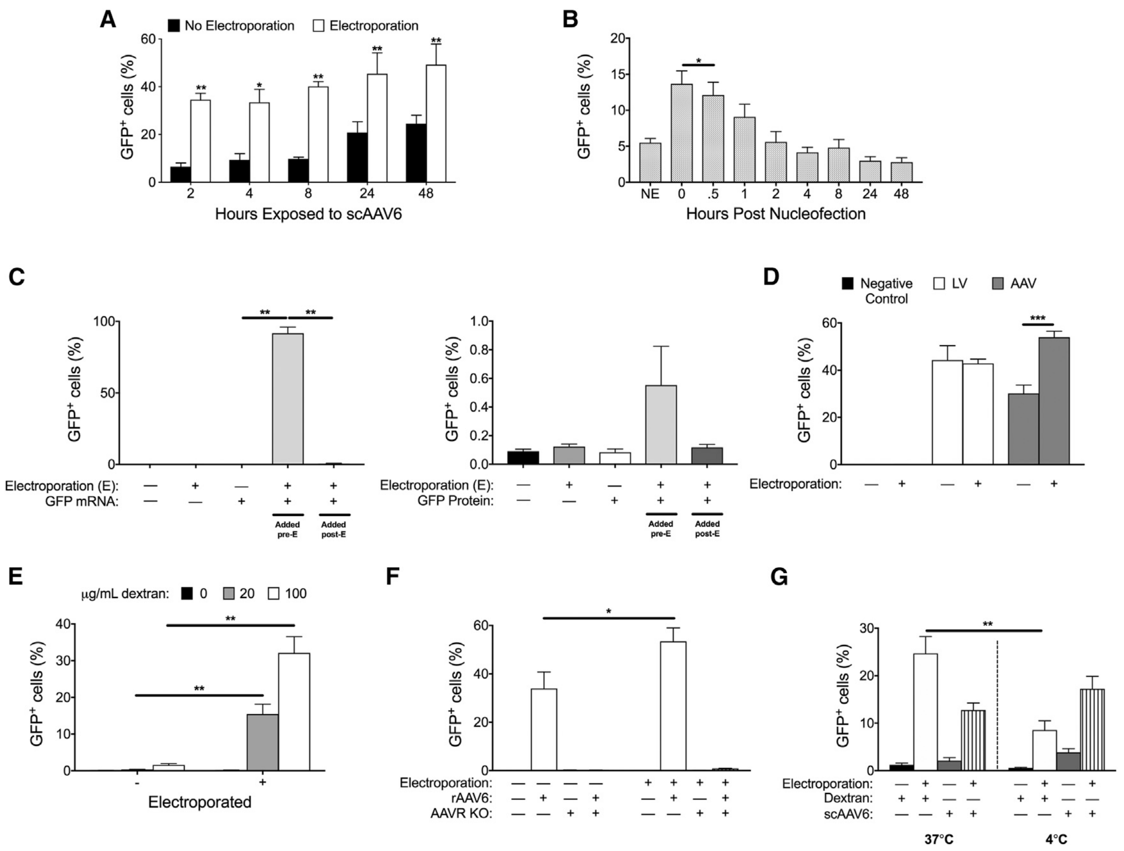

Figure 2. Electroporation Significantly Increases Endocytosis, Aiding Transduction of rAAV6 through the AAVR Pathway

(A) Percentage of CB CD34+ cells that are GFP$^+$ 48 hr after transduction with scAAV6 of cells that are either electroporated or left un-electroporated. scAAV6 was washed off after varying durations of exposure. **p < 0.01; *p < 0.05 two-way ANOVA and Bonferroni's multiple comparison test. (B) Percentage of K562 cells that are GFP$^+$ when scAAV6 is added at different time points post-electroporation (N = 3 replicates). *p < 0.05; paired t test. (C) Percentage of K562 cells that are GFP$^+$ (n = 3 replicates) following delivery of GFP mRNA (left panel, 0.8 μg/1 × 10^5 cells) GFP protein (right panel, 2.5 μg/1 × 10^5 cells) without, before, and after electroporation. **p < 0.01; paired t test. (D) Percentage of K562 cells that are GFP$^+$ 48 hr post-transduction with a lentivirus (LV) or scAAV6 donor when cells are electroporated versus not electroporated (n = 2–6 replicates). ***p < 0.01; paired t test. (E) Percentage of GFP$^+$ K562 cells following delivery of 1 × 10^4 MW dextran coupled to CF488A with or without electroporation. All treatment groups were washed 3× prior to analysis. Dextran-CF488A endocytosis was measured by flow cytometry (n = 3 replicates). **p < 0.01; paired t test. (F) Percentage of cells that are GFP$^+$ when wild-type or AAVR KO K562 cells are transduced with rAAV6 with and without electroporation (n = 3 replicates). *p < 0.05; paired t test. All values are means, and error bars represent SEM. (G) Percentage of GFP+ CB CD34$^+$ cells after application of scAAV or dextran when cells were at 4°C or 37°C and were electroporated or non-electroporated (n = 2 CB CD34$^+$ donors; **p < 0.01; unpaired t test).

by two hr post-electroporation (p = 0.03). We first hypothesized that the boost in transduction efficiency could be due to electroporation causing long-lasting increased porosity of the cell membrane that allows for the entry of exogenous large molecules into the cell. If this were the case, then mRNA and proteins that are frequently electroporated into cells should also be able to enter cells if added after electroporation. However, electroporation of K562 cells immediately followed by addition of GFP-encoding mRNA (p = 0.003) or GFP protein to the cells did not boost uptake (Figure 2C).

We next sought to determine whether the EAT effect was unique to rAAV6 or a phenomenon that occurred with other viruses as well. We compared the transduction efficiency of scAAV6 to that of vesicular stomatitis virus G protein (VSV-G)-pseudotyped lentiviral vectors (LVs) when K562 cells had or had not undergone electroporation immediately prior to transduction. Whereas electroporation increased rAAV6 transduction, it did not significantly increase transduction of cells by LVs (Figure 2D). Because the transduction pathways of rAAV6 and VSV-G-pseudotyped LVs differ (AAV6 transduction, unlike lentivirus, requires endocytosis), we next assessed whether electroporation increased cellular endocytosis.[27–30] We did so by assessing the uptake of fluorescent dextran in K562s, which is commonly used to measure endocytosis in cells.[31,32] When adding Alexa-488-conjugated dextran to cells for twenty minutes that had or had not been electroporated, we found that electroporation significantly increased uptake of dextran at concentrations of 20 μg/mL and 100 μg/mL (p = 0.005; p = 0.002; Figure 2E).

An essential receptor for AAV uptake was recently described.[33] To investigate whether the uptake of AAV6 was through the AAV

receptor (AAVR) pathway, we next attempted to transduce an AAVR knockout (KO) K562 cell line with scAAV6 immediately after electroporation (Figure 2F). We found that K562 AAVR KO cells could not be transduced well by scAAV6 under non-electroporated conditions or when scAAV6 was added immediately after electroporation, demonstrating that the EAT effect occurs through an AAVR-dependent pathway.[33]

Having found that electroporation did increase endocytosis of dextran and that the EAT effect required AAVR for AAV6 uptake into cells, we attempted to inhibit the EAT affect by keeping cells at 4°C, a common method for inhibiting endocytosis.[34,35] Whereas we found that we were able to significantly reduce endocytosis of dextran when CB CD34$^+$ cells were both electroporated and non-electroporated by keeping cells at 4°C, we found that we were unable to inhibit the uptake of rAAV6 by keeping cells at 4°C both when cells were electroporated and non-electroporated ($p = 0.0025$; Figure 2G). This is likely due to dextran entering cells through fluid phase endocytosis, unlike rAAV6, which enters through AAVR-receptor-mediated endocytosis.[33,36]

Together, these data demonstrate that electroporation has a substantial effect on transduction of AAV6 into cells through the AAVR pathway, potentially due to electroporation increasing the amount of endocytosis undergone by cells. However, we found that AAV6 endocytosis was refractory to inhibition by 4°C culture conditions (even in the absence of electroporation). Further work is required to fully characterize the mechanism by which electroporation increases AAV6 transduction into cells via the essential AAVR.

Optimizing rAAV6 Donor Delivery to Achieve Consistently High Levels of HR

We next optimized rAAV6 donor delivery by titering the MOI or vector genomes/cell (vgs/cell) that stimulated the highest rates of HR in HSPCs with minimal amounts of cellular toxicity (Figure 3A). Following delivery of Cas9/sgRNA HBB-RNP to cells by electroporation, CB HSPCs were transduced with rAAV6-SFFV-GFP donor[15] at MOIs ranging from 1×10^4 to 2×10^5 vgs/cell. We found that rates of HR significantly increased with increasing MOI (Figure 3B, left panel) but started to plateau at an MOI of 1×10^5. Because clinical-grade rAAV6 is costly and laborious to produce, we deemed that the added boost in HR between 1×10^5 and 2×10^5 vgs/cell ultimately was not great enough to justify using an MOI of $>1 \times 10^5$ to stimulate HR in HSPCs. Additionally, we found no significant difference in cell viability across all MOIs (Figure 3B, right panel).

Previous research has shown that, when a transgene delivered using a rAAV6 vector is integrated into the genome by HR, there is a marked increase in transgene expression that allows for the identification and enrichment of edited engraftable HSPCs.[5] To identify clinically relevant human promoters that allow for the highest frequency enrichment of *HBB*-targeted HSPCs, we compared the effectiveness of several promoters at driving GFP expression once integrated into the *HBB* locus. We constructed several different donors, each containing the same arms of homology to the β-globin gene but with GFP expression driven by the following different promoters: β-actin, elongation factor one alpha (EF1α), elongation factor 1 alpha short (EFs), phosphoglycerate kinase (PGK), ubiquitin C (UBC), and the positive control, spleen focus-forming virus (SFFV) (Figure 3C). When electroporating CB HSPCs and transducing them with each of these rAAV6 vectors in the absence of Cas9 RNP, we found that all of the human promoters drove lower levels of GFP expression episomally compared to the SFFV promoter (Figure 3D). Introducing the Cas9/sgRNA nuclease along with our rAAV6 donors, the percentage of GFP^{high} cells varied dramatically depending on the promoter used (Figure 3D). We found that the highest numbers of measured integration events were achieved using the SFFV promoter, with an average of 32% cells that underwent HR, as detected by GFP^{high}. The clinically relevant human promoters that yielded the most comparable expression were the PGK and UBC promoters with an average of 27% and 24% GFP^{high}, respectively (Figures 3D and 3E). All of the other human promoters resulted in less than half as many edited cells detectable by GFP^{high} compared to the SFFV promoter. The SFFV promoter also drove significantly higher GFP expression in all cells with integration as measured by mean fluorescence intensity (MFI), at almost a log-fold higher MFI than any other human promoter (Figures 3D and 3E). Interestingly, each of the promoters gave a unique GFP profile in cells on a fluorescence-activated cell sorting (FACS) plot, with the UBC promoter resulting in the most distinct population of targeted cells (Figure 3D). To determine whether the promoters were an accurate measure of HR frequencies, we performed digital droplet PCR (ddPCR) to quantify the number of targeted alleles in the bulk population (Figures 3F and S4). We found no significant difference between the percentages of alleles that underwent HR between any of the different constructs by ddPCR, with all homologous donors mediating greater than 12% allelic-targeted integration into *HBB* (Figure 3F).

Low-Density Culture Conditions prior to Gene Targeting Expand Human LT-HSCs, Leading to Greater HR Frequencies

It has been previously demonstrated that a two-day cytokine stimulation before gene targeting significantly increased the percentage of repopulating LT-HSCs that undergo HR.[11] A large body of work has also demonstrated that the cell cycle status of cells plays a crucial role in the choice of HR versus NHEJ in DSB repair.[37–40] Most LT-HSCs in the bone marrow remain quiescent, with only 5% of these cells moving through S/G_2/M phases of the cell cycle at any given time,[41,42] and are thus skewed toward NHEJ-mediated DSB repair.[43] We hypothesized that if we could stimulate HSPCs to enter the cell cycle and expand before gene targeting, then higher frequencies of HR could be achieved. It has been shown that a simple way to expand HSPCs *ex vivo* was by plating HSPCs at low densities using a fed-batch media dilution approach, and possibly even more effective is when this approach is combined with the LT-HSC expansion small molecules, UM171 and SR1.[44,45]

To that end, both CB and PB HSPCs were plated at concentrations of 1×10^6 cells/mL, 5×10^5 cells/mL, and 1×10^5 cells/mL and cultured

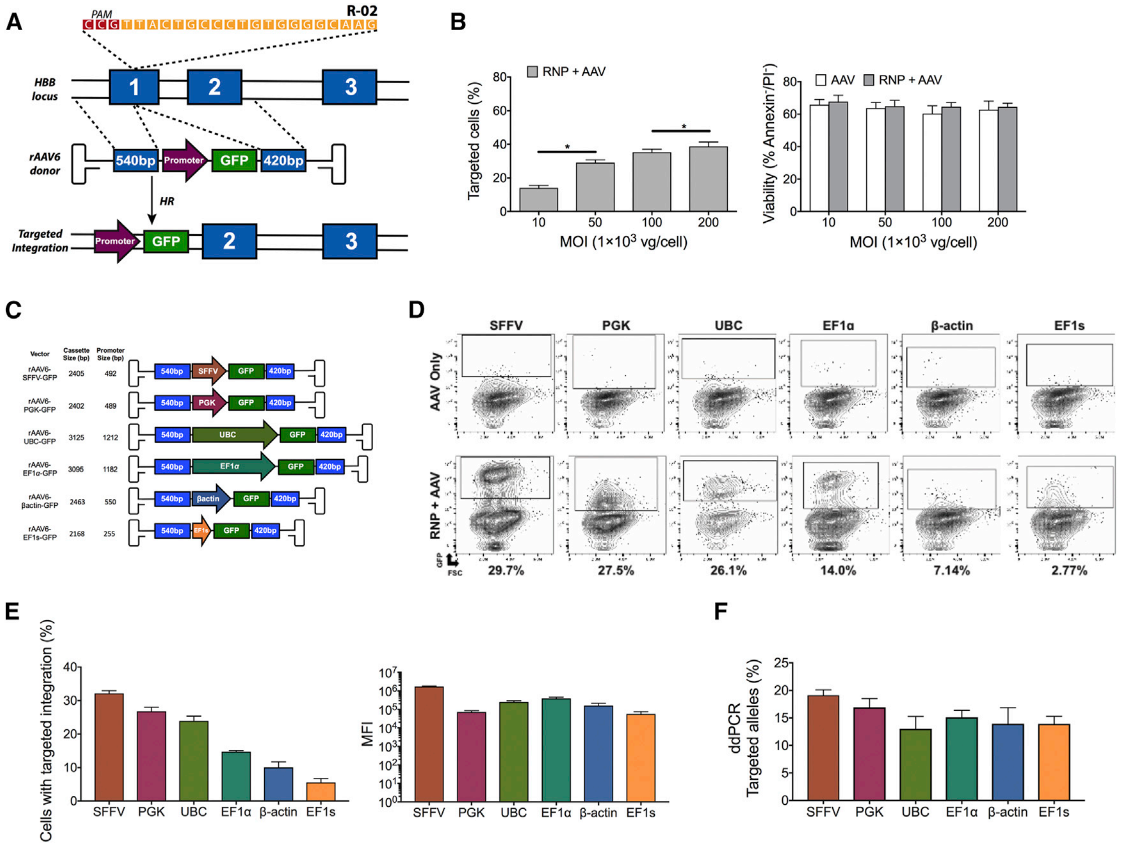

Figure 3. Optimization of Transgene Promoter Expression to Maximize Efficiencies of HR as well as Detection and Enrichment of Edited HSPCs

All experiments in this figure were performed on cord-blood-derived $CD34^+$ cells. (A) Schematic outlining the gene-editing process at the *HBB* locus following DSB initiation by Cas9/sgRNA and HR using a *HBB*-specific single-stranded AAV6 containing SFFV-GFP (ssAAV6-SFFV-GFP). (B) Percentage of targeted cells (as measured by GFP^{high}, left panel) and cell viability 24 hr post-targeting (as measured by annexin V and P.I. staining, right panel) when cells are transduced with rAAV6 at various MOIs. $^*p < 0.05$; paired t test. (C) Schematic depicting rAAV6 constructs homologous to the *HBB* locus, which were used to target HSPCs. GFP expression is driven by various promoters (MOI = 5×10^4; n = 4 $CD34^+$ donors). $^*p < 0.05$; paired t test. (D) Representative FACS plots depicting the gating scheme for GFP^+ cells targeted with the various rAAV6-*promoter*-GFP constructs 4 days post-electroporation (MOI = 5×10^4). Top panels display cells transduced with rAAV6 donors after mock electroporation (no Cas9/sgRNA), and bottom panels display cells with rAAV6 donor vector transduction after electroporation of Cas9/sgRNA RNP measured 4 days post-electroporation (MOI = 5×10^4). (E) Left panel shows the percentage of cells that have undergone HR as detected by the frequencies of GFP^{high} cells 4 days post-electroporation. Right panel shows the mean fluorescent intensity (MFI) of cells that have undergone HR with each of the different promoter constructs (MOI = 5×10^4). (F) Number of *HBB* alleles in the targeted HSPC population that have undergone HR as measured by ddPCR (n = 3 $CD34^+$ donors) with gDNA harvest 4 days post-electroporation. All values are means, and error bars represent SEM.

for two days and then were targeted for HR at *HBB*. It was found that the lower cell density culture conditions led to a significant expansion of cells in culture. Plating cells at $<1 \times 10^6$ cells/mL resulted in significantly greater HSPC expansion in culture with cells cultured at 1×10^6 cells/mL expanding 1.5-fold, cells plated at 5×10^5 cells/mL expanding 2-fold, and cells plated at 1×10^5 cells/mL expanding 3-fold (1×10^6 cells/mL versus 5×10^5 cells/mL, p = 0.0007; 5×10^5 cells/mL versus 1×10^5 cells/mL, p = 0.007; Figure 4A). We also found that cells that were plated at lower densities had a higher percentage of cells that underwent HR (Figure 4B). When the fold expansion of cells was plotted against the percentage of cells that underwent HR on a linear regression scatterplot, a clear positive correlation between cell expansion and HR frequencies was observed ($R^2 = 0.36$; p = 0.0001; Figure 4C). Furthermore, when donors were sub-plotted individually, an even more statistically significant correlation was apparent (average R^2 = 0.83; Figure S5). This demonstrated a clear trend between the expansion of cells in culture before gene targeting and the percentage of cells that underwent HR, which was $CD34^+$ cell source (CB and PB) and donor independent.

To rule out that increased targeting efficiencies at lower density were due to more DSBs or increased availability of gene targeting reagents, we compared the kinetics of HR in CB HSPCs plated at different cells densities. HSPCs were targeted with the Cas9/sgRNA RNP at a concentration of 300 μg/mL, and cells were transduced at an MOI of 5×10^4 with rAAV6-SFFV-GFP. Tracking the frequency at which indels formed in HSPCs over time, we found no major differences

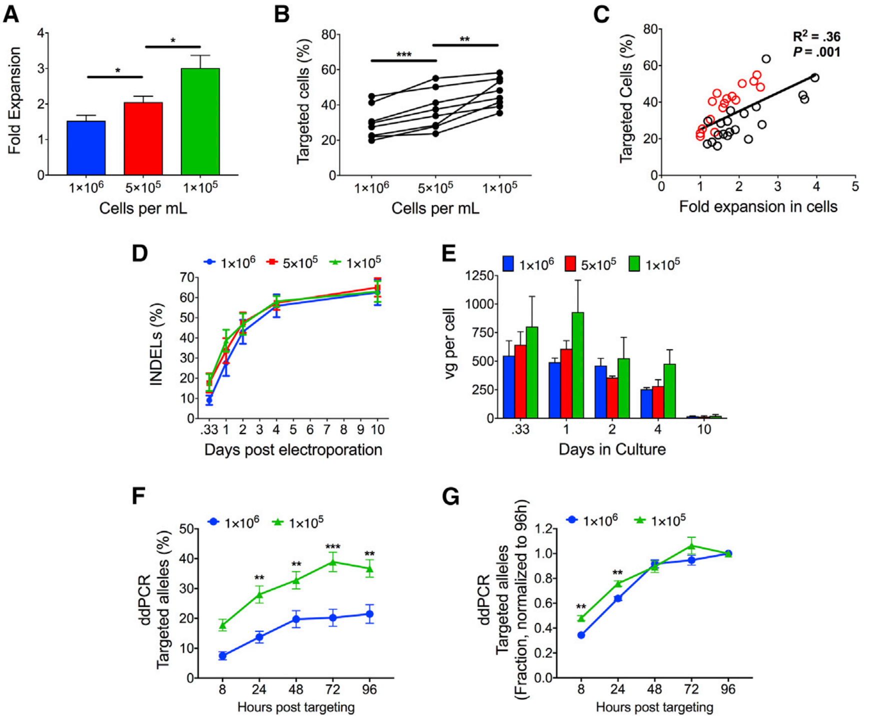

Figure 4. Low-Density Culture Conditions Significantly Increase HR Frequencies in HSPCs *In Vitro*

(A) Fold increase in the number of HSPCs present in culture when cells are plated at 1×10^6, 5×10^5, and 1×10^5 cells/mL in culture (n = 6 both cord blood and mobilized $CD34^+$ donors used). *$p < 0.05$; paired t test. (B) Percentage of cells that have undergone HR when plated at different densities. Each line represents a single donor targeted after two days of culture at 1×10^6, 5×10^5, and 1×10^5 cells/mL (n = 8 both cord blood and mobilized $CD34^+$ donors were used). ***$p < 0.001$; **$p < 0.01$; paired t test. (C) Scatterplot of the percent of HSPCs that have undergone HR plotted against the fold expansion of cells after two days in culture prior to editing, red dots indicate mobilized peripheral blood donors, and black dots indicate cord blood donors (n = 36 $CD34^+$ donors). (D) Percentage of alleles with an indel over time when HSPCs are cultured at different densities prior to targeting (n = 5 or 6 CB $CD34^+$ donors). (E) Number of vector genomes delivered by rAAV6 as measured by inverted terminal repeat (ITR) qPCR relative to an albumin standard when HSPCs are cultured at different densities prior to targeting is shown (n = 3 or 4 CB $CD34^+$ donors). (F) Comparison of the percentage of CB CD34+ cells targeted over time between cord blood CD34+ cells cultured at a density of 1×10^6 cells/mL or 1×10^5 cells/mL (n = 4 $CD34^+$ donors). **$p < 0.01$; ***$p < 0.001$; two-way ANOVA and Bonferroni's multiple comparison test. (G) Data in (F) normalized to 96 hr are shown. **$p < 0.01$; paired t test. All values are means, and error bars represent SEM.

across samples (Figure 4D). Monitoring rAAV6 transduction of HSPCs by qPCR of the vector genome relative to an albumin standard, we found no significant difference between plating conditions and number of vgs per cell over time; however, we did observe a positive trend in the number of vgs/cell with low-density culture conditions 8 and 24 hr post-transduction (Figure 4E). On average, each cell received approximately 500 vgs, and transduction peaked after 8 hr in culture. Viral genomes rapidly diluted out of cells over time, with less than 5% of the total vectors present at 10 days post-transduction compared to 24 hr post-transduction (Figure 4E).

With no major differences in the kinetics of indel formation and transduction between culturing conditions, we next compared the kinetics of HR over time when CB HSPCs cells were plated at low density compared to high density prior to targeting. Cells were plated at either 1×10^6 cells/mL or 1×10^5 cells/mL two days prior to targeting, and the percentage of targeted alleles at the HBB locus was measured over time by ddPCR. As expected, there were more alleles targeted when cells were cultured at 1×10^5 cells/mL at each time point (Figure 4F). When normalized to the total amount of targeting that occurred by 96 hr post-electroporation, we found that there was only a small but statistically significant increase in the frequency at which alleles were targeted when cells were plated at 1×10^5 cells/mL and that this increase in the rate of targeting was only present within the first 24 hr after targeting (8 hr time point, p = 0.0007; 24 hr time point, p = 0.007; Figure 4G). Overall, low-density cell culture conditions significantly increase HR frequencies in HSPCs, presumably due to an increase in the number of HSPCs cycling prior to Cas9/sgRNA/rAAV6 targeting rather than by an alteration to the kinetics of HR or NHEJ.

Whereas the low-density cell culture conditions increased the percentage of targeted HSPCs, it was possible that this increase in targeting is more prominent in short-lived progenitors compared to LT-HSCs. To test whether low-density cell culture conditions increased the frequency of gene targeting in LT-HSCs, we compared the ability of gene-edited CB HSPCs to engraft into immunodeficient mice when they were cultured at 1×10^5 cells/mL or 1×10^6 cells/mL for two days prior to targeting. Culturing cells at the different densities for two days prior to targeting, we again saw an increase in the percentage of HSPCs edited when cells were cultured at 1×10^5 cells/mL (53.5%) compared to when they were cultured at 1×10^6 cells/mL (40.3%; Figure 5A). After 6 total days in culture (2 before targeting and 4 after targeting), we then transplanted by intravenous injection an equivalent number of bulk edited cells (300,000 cells) that were treated from each group into immunodeficient mice and then analyzed mice bone marrow for engraftment of human cells 8 and 16 weeks later (Figure 5B). We found that there was no significant difference in engraftment of human cells in mice between the two conditions (Figure 5B). Both culture conditions gave rise to bi-lineage reconstitution of the hematopoietic system in NSG mice (Figures 5C and 5D). Importantly, after 16 weeks in

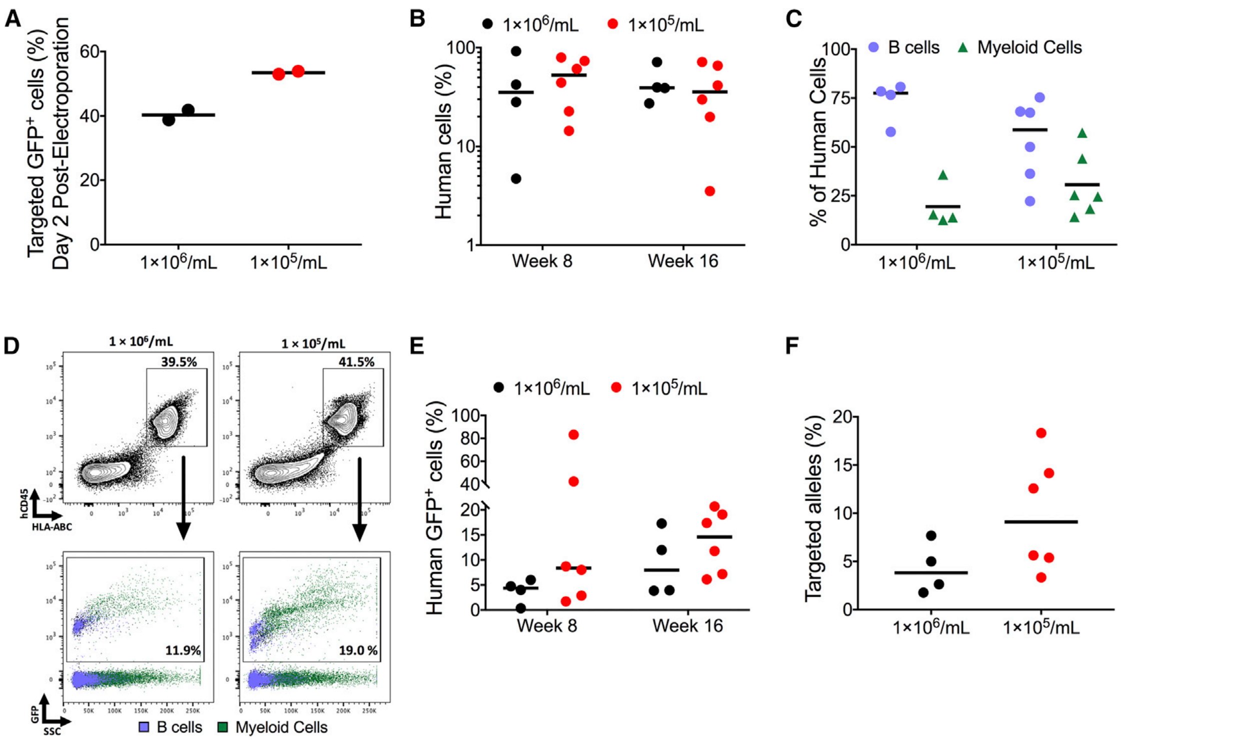

Figure 5. Low-Density Cell Culture Conditions Primes Multi-lineage Repopulating HSPCs for HR

All experiments in this figure were performed on cord-blood-derived CD34+ cells. (A) Median percentage of cells that have undergone HR *in vitro* two days after electroporation while plated at two different culture densities is shown (n = 2 cord blood donors). (B) Median percentage of human engraftment ($hCD45^+$/$HLA\text{-}ABC^+$) in NSG mice after intravenous transplantation of cells cultured at the two different culture densities, with analysis at week 8 and week 16. Assessment at week 8 was performed by gathering bone marrow aspirates, and at week 16, mice were harvested (n = 4 mice for 1×10^6/mL culture density; n = 6 mice for 1×10^5/mL culture density). (C) Median percentage of B cells ($CD19^+$) and myeloid cells ($CD33^+$) of the human cell population harvested from week 16 NSG mice. (D) Representative FACS plot of mouse bone marrow 16 weeks post-transplantation of cells cultured in respective culture densities. Top: representative FACS plot of human engraftment ($hCD45^+$/$HLA\text{-}ABC^+$) in NSG mice. Bottom: representative FACS plot showing GFP-expressing human cells. $CD19^+$ B cells and $CD33^+$ myeloid cells are back gated and shown in purple and green, respectively. (E) Median percentage of GFP-expressing human cells. Of the human cells, GFP^+-targeted cells are indicated by a MFI shift as seen in Figure 4D. Paired t test of the dataset resulted in no statistical significance with $p > 0.05$. (F) Number of *HBB* alleles in the targeted LT-HSC population 16 weeks after transplantation into NSG mice that have undergone HR as measured by ddPCR. Paired t test of the dataset resulted in no statistical significance with $p > 0.05$. All error bars are the standard error between replicates.

mice, 14.6% of engrafted human cells were gene edited from cells that were cultured at 1×10^5 cells/mL compared to 7.97% when cells were cultured at 1×10^6 cells/mL (Figures 5D and 5E). When we performed ddPCR on cells harvested after 16 weeks in immunodeficient mice to detect the frequency of cells with edited alleles, we found that cells cultured at 1×10^5 cells/mL had 9.1% of alleles edited compared to 3.8% when cells were cultured at 1×10^6 cells/mL (Figure 5F). The higher ratio of engrafted targeted alleles to targeted cells when HSPCs were cultured at 1×10^5 cells/mL compared to 1×10^6 cells/mL suggests that low-density cell culture conditions also increases the frequency of bi-allelic targeting in LT-HSCs. These data suggest that low-density cell culture conditions lead to higher frequencies of targeting in durable, repopulating HSPCs.

Ex Vivo Expansion of *HBB* Gene-Targeted LT-HSCs

It has been reported that repopulating LT-HSCs can be expanded in culture for up to fourteen days using similar low-density cell culture conditions combined with UM171 and SR1.[44,46] We tested the hypothesis that FACS-enriched, *HBB*-targeted repopulating LT-HSCs could be expanded by keeping cells in culture with UM171/SR1 under low-density culture conditions. To that end, HSPCs were targeted with rAAV6-UbC-GFP and sorted for targeted cells (and the CD34 cell surface marker) four days post-electroporation and then either transplanted into mice or cultured for an additional 8 days. They were then again sorted for GFP^{high} and $CD34^+$ cells (Figures 6A and S6). We found significant donor-to-donor variability in terms of the percentage of targeted cells, with a median of 37.3% of cells edited as measured by GFP^{high} cell frequencies at 4 days post-electroporation (Figure 6B).

To test the engraftment potential of expanded $CD34^+$/GFP^{high} HSPCs on a per cell basis, we transplanted an equivalent cell dose of CB $CD34^+$/GFP^{high} cells intrafemorally into NSG mice on day 4 (6 days total culture time) or day 12 (14 days total culture time)

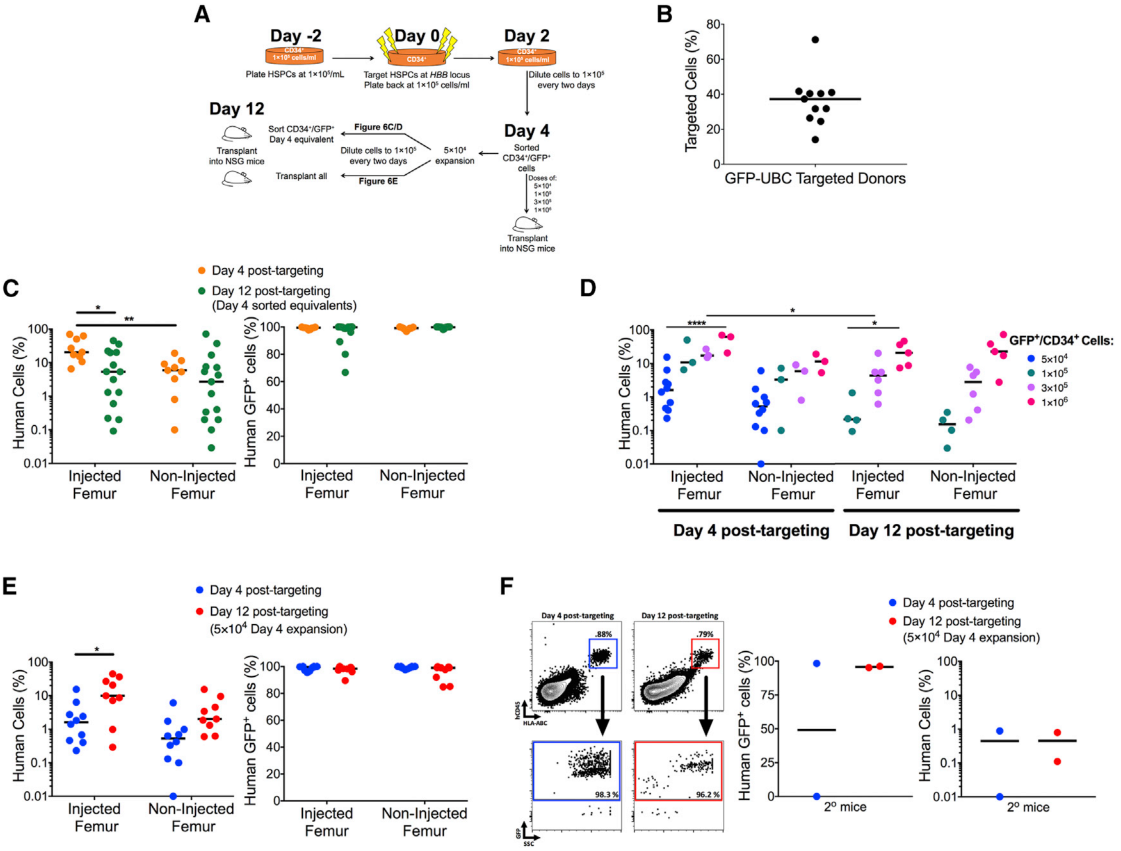

Figure 6. A Pure Population of Gene-Edited HSPCs Can Be Expanded in Low-Density Culture in Presence of SR1 and UM171

All experiments in this figure were performed on cord-blood-derived CD34+ cells. (A) Schematic showing the timeline for editing and expansion of HSPCs in culture. (B) Median percentage of cells targeted using AAV-UBC-GFP (n = each data point represents a unique donor). (C) Left: percentage of human cell engraftment of gene-edited CD34$^+$/GFP$^+$ HSPCs at 16 weeks after intrafemoral transplant of an equivalent number of cells following 4 or 12 days post-targeting. 5×10^4, 1×10^5, 3×10^5, and 1×10^6 CD34$^+$/GFP^{high} HSPCs were transplanted into NSG mice (day 4 post-targeting transplants: n = 19 mice; day 12 post-targeting transplants: n = 15 mice). **$p < 0.01$, paired t test; *$p < 0.05$, unpaired t test. Right: median percentage of GFP-expressing human cells 16 weeks post-transplant following 4 or 12 days post-targeting. The genome-editing frequencies for day 4 and 12 ($\leq 1 \times 10^6$) cells are also reported in Table 1. (D) Data are the same as presented in (C) but are now displayed by CD34$^+$/GFP^{high} cell doses showing a dose-response of engraftment. The genome editing frequencies for these mice are shown in Table 1. The 5×10^4 cell dose transplanted group is the same one presented in (E). *$p < 0.05$, paired t test; ****$p < 0.0001$, paired t test. (E) Left: percentage of human engraftment in NSG mice after intrafemoral transplantation of 5×10^4 CD34$^+$/GFP$^+$-sorted HSPCs 4 days post-targeting compared to transplantation of 5×10^4 CD34$^+$GFP$^+$-sorted HSPCs that were expanded from day 4 to 12 (n = 6 CD34$^+$ donors; n = 10 day 4 mice; n = 9 day 12 mice). *$p < 0.05$; unpaired t test. Right: median percentage of GFP-expressing human cells 16 weeks post-transplant following 4 or 12 days post-targeting. The genome-editing frequencies for day 12 ($\geq 2 \times 10^6$ cells) are also reported in Table 1. (F) Left: representative FACS plots depicting the gating scheme for engrafted human cells (hCD45$^+$/HLA-ABC$^+$) in secondary (2°) NSG mice after intrafemoral transplantation of 5×10^4 CD34$^+$/GFP$^+$-sorted HSPCs 4 days post-targeting compared to transplantation of 5×10^4 CD34$^+$GFP$^+$-sorted HSPCs that were expanded from days 4 to 12. The lower FACS panels show the percentage of GFP$^+$ human cell derived from the human cell population. Middle: the median percentage of human-GFP$^+$-targeted cells from 2° NSG mice. Right: the median percentage of human engraftment from 2° NSG mice after intrafemoral transplantation (n = 2 day 4 mice, n = 2 day 12 mice). All error bars are the standard error between replicates.

post-targeting. We found, as others have reported,[44–46] that the equivalent numbers of CD34$^+$ HSPCs lost stem cell potential over time in culture on a per cell basis ($p = 0.0169$; Figure 6C). Mice transplanted with equivalent numbers (either 1×10^5, 3×10^5, or 1×10^6) of *HBB*-targeted CD34$^+$/GFP$^+$ HSPCs from day 6 displayed a median of 20.5% human chimerism in the injected femur, significantly greater than the 5.9% human chimerism found in the non-injected femur (Figures 6C and 6D; $p = 0.0078$). In comparison, the median human chimerism of mice transplanted with equivalent numbers of CD34$^+$ HSPCs from day 14 was 5.3% and 2.7% in the injected and non-injected femurs, respectively (Figure 6C). These data are corroborated by the observation that *HBB*-targeted HSPCs cultured for 12 days had a lower fraction of cells expressing markers, like CD90 and EPCR (Figure S6).[47] Importantly though, there was a cell dose-dependent increase in the percent human chimerism when transplanting a pure population of both non-expanded ($p = 0.0002$; 5×10^4 versus 1×10^6) and expanded ($p = 0.0474$; 1×10^5 versus 1×10^6) gene-targeted cells (Figure 6D). As previously seen, HSPCs expanded for

Table 1. Outline of the NSG Mice with Multi-lineage Engraftment Transplanted with *HBB*-Targeted HSPCs

Time when Transplanted HSPCs (Post-targeting)	No. of Mice Transplanted	No. of Mice with Bi-lineage Engraftment	Percentage of Mice with Bi-lineage Engraftment	B Cell Range in Mice with Bi-lineage Engraftment	Percent GFP$^+$ Human Cells (Median)
Day 4	9	6	66.6%	10%–75%	99.4%
Day 12 ($\leq 1 \times 10^6$ cells)	15	2	13.3%	4%–42%	99.8%
Day 12 ($\geq 2 \times 10^6$ cells)	9	4	44.4%	3%–45%	99.0%

This table summarizes the time and the amount of *HBB*-targeted HSPCs that were transplanted into NSG mice via the intrafemoral route. 16 weeks post-transplant, bone marrow was harvested from femurs, and human cell chimerism (huCD45/HLA-ABC), along with B cell (CD19$^+$) and myeloid cell (CD33$^+$) lineage reconstitution, was evaluated. Genome-editing frequencies were calculated via GFP expression within the human cell populations. These data demonstrate that expanded *HBB*-targeted HSPCs have reduced lymphoid reconstitution (with the rest of the cells identified as CD33$^+$) due to the enhanced cycling of short-lived progenitors (see Figures 6C and 6D), but this can be partially rescued by injection of greater numbers of *HBB*-targeted HSPCs.

12 days post-targeting had fewer mice with bi-lineage engraftment at similar doses (Table 1).[45] Importantly though, the vast majority of human cells were gene edited (GFP$^+$; 96.7%–100% of the human cells; Figure 6C; Table 1). This information implies that, in the *HBB*-targeted CD34$^+$ fraction, the short-lived myeloid repopulating progenitors are cycling more frequently than LT-HSCs over two weeks in low-density culture conditions with UM171/SR1.

Whereas we have shown that equivalent numbers of *HBB* gene-edited CD34$^+$ HSPCs have less repopulating potential after 12 days in culture compared to 4 days in culture, we hypothesized that we could rescue the decreased engraftment by transplanting a greater number of edited cells. To test this hypothesis, we FACS-sorted CB CD34$^+$/GFP^{high} cells after four days in culture (post-targeting), and then these sorted cells were split into two samples of 5×10^4 cells each: one of the samples was immediately transplanted intrafemorally into NSG mice (n = 10 mice) and the remaining sample was plated and cultured at 1×10^5 cells/mL in UM171/SR1 supplemented media for an additional 8 days before transplantation. From days 4 to 12 post-targeting, the 5×10^4 CD34$^+$/GFP^{high} cells expanded into $\sim 2.9 \times 10^6$ (mean fold increase = 57.5; n = 6 CD34$^+$ donors) cells and then the entire population of cells was transplanted into NSG mice (n = 9 mice). Analyzing human chimerism of mice transplanted with gene-edited HSPCs 16 weeks post-transplantation, we found that the mice transplanted with $\geq 2 \times 10^6$ cells from day 12 displayed a 5.4-fold higher engraftment compared to the ten mice transplanted with 5×10^4 cells from day 4, with concurrent increased engraftment in the contralateral femur ($p = 0.0152$ injected femur; $p = 0.07$ non-injected femur; Figure 6E). In contrast to NSG mice transplanted with less than 2×10^6 expanded *HBB*-targeted HSPCs, 44% of NSG mice transplanted with greater than 2×10^6 expanded *HBB*-targeted HSPCs displayed lineage reconstitution of B cells (Table 1). As in our previous transplants, $\geq$99.0% of engrafted human cells were gene edited (Figures 6E and S7; Table 1). Importantly, secondary transplantation experiments showed that expanded and unexpanded HBB-targeted HSPCs maintained long-term repopulation capacity (Figure 6F). Collectively, these data demonstrate that low-density culture conditions combined with UM171/SR1 could expand and maintain gene-edited LT-HSCs, albeit to a much lower frequency than progenitors, to achieve long-term bi-lineage engraftment of a very pure gene-edited hematopoietic population.

DISCUSSION

In this study, we present an optimized protocol for using the Cas9/sgRNA system in combination with rAAV6 homologous donor vectors to edit and expand HSPCs that have been targeted at the *HBB* locus. We demonstrate the following key findings: (1) use of a *HBB* Cas9/sgRNA protocol that induces DSBs in LT-HSCs that can give rise to bi-lineage engraftment in serial transplants, (2) EAT of rAAV6, (3) the number of vector genomes of rAAV6 necessary for high levels of gene targeting as well as promoter activity in HSPCs, (4) that low-density culture conditions prime LT-HSCs for HR, and (5) that *HBB* gene-edited LT-HSCs can be expanded using low-density culture conditions combined with UM171 and SR1.

We found that delivering the wild-type Cas9/sgRNA system (derived from *Streptococcus pyogenes*) as an RNP (150–300 μg/mL) with an MS-modified guide RNA creates high-efficiency indels at the *HBB* locus in repopulating LT-HSCs. Following transplantation into NSG mice, we found no significant difference in the amount of indels created in LT-HSCs compared to other cell types, as evident by equal frequencies of indels in secondary transplant recipients. It should be noted that there was a 2-fold reduction in the amount of indels present in engrafted cells following primary transplantation into NSG mice. Whereas a reduction in generating indels in LT-HSC has been reported (in the autologous setting with editing of nonhuman primate LT-HSCs),[48] this is potentially due to (1) unedited progenitors outcompeting edited progenitors in primary transplants, an artifact which no longer persisted following secondary transplantation into NSG mice, and/or (2) enrichment of edited LT-HSCs in the secondary transplants. Nevertheless, these results demonstrate *HBB*-directed Cas9/sgRNA RNPs can elicit a significant amount of DSBs in LT-HSC, which is essential for promoting HR.

In the process of optimizing delivery of our rAAV6 donor, we discovered that electroporation improves rAAV6 transduction efficiencies, which we have termed EAT. EAT explains why adding rAAV6 donor immediately following electroporation has proven to be the most effective at stimulating HR in hematopoietic cells, both HSPCs and K562 cells. This is a finding that our group and others have also demonstrated.[5,14,25] Building upon this previous work as well as our findings, we performed a series of experiments to identify the mechanism of action of EAT.

We found that electroporation did not cause a ubiquitous increase in the uptake of macromolecules added post-electroporation by cells, with no significant increase of GFP mRNA uptake, GFP protein uptake, or lentiviral transduction. Dextran, a commonly used reagent to study cellular endocytosis, increased in uptake after electroporation. We were not able to recapitulate the EAT effect in AAVR KO K562 cells, demonstrating the importance of the AAVR receptor. These data suggest that the EAT effect is a result of increased endocytosis by cells following electroporation and would explain why electroporation does not significantly increase transduction of cells by VSV-G-pseudotyped LVs, as the transduction pathway of LVs, unlike AAV, is thought to be independent of endocytosis.[27,28,30] We blocked endocytosis by culturing cells at 4°C following electroporation and found that, whereas we were able to inhibit dextran endocytosis in both electroporated and non-electroporated cells with 4°C cell culture, we were unable to block AAV6 uptake in either context with a 4°C cell culture. This is likely due to the different endocytic mechanisms by which AAV6 and dextran enter cells, with AAV6 being receptor mediated. Together, these data strongly suggest that the EAT effect is due to electroporation causing significantly more endocytosis by cells; however, further studies are required to elucidate the underlying molecular mechanism and also open up the possibility of using electroporation to increase endocytosis of other molecules.

The observation that electroporation significantly increases AAV6 transduction into cells has significant value for both *ex vivo* and *in vivo* gene therapy. For any *in vitro* work, our data and work from other groups clearly demonstrate that the best time to deliver rAAV6 into cells for *in vitro* genome editing is after electroporation.[14,25] Furthermore, whereas it is impractical to electroporate a patients organ *in vivo* to increase AAV transduction, if the underlying mechanisms behind the EAT effect can be elucidated and manipulated to recapitulate this effect with less disruptive treatment, it opens a potential avenue to increase the effectiveness of AAV-related therapies. For example, if the 5-fold increase in the percentage of cells that undergo HR when AAV6 is added after electroporation (due to better transduction of cells with the AAV vector) that we have found here can be recapitulated for *in vivo* genome editing therapies, it could significantly boost the clinical efficacy of therapies that rely on AAV as a vector for delivery, such as the *in vivo* genome-editing therapy currently in clinical trials to treat mucopolysaccharidosis type II.[49]

We also provide the first comparison of the ability of a variety of constitutive clinically relevant promoters to drive expression of an integrated transgene at the *HBB* locus in HSPCs. Previous studies have compared the effectiveness of different promoters randomly integrated throughout the genome in HSPCs using LVs or have used a single promoter, such as MND or PGK, to drive the expression of a transgene introduced into the genome by HR.[11,14,15,50–52] Our study has the advantage of testing a variety of different promoters within a single locus, eliminating any variability caused by random insertion into the genome. We saw significant variation in the activity of different clinically relevant promoters tested to drive GFP expression in CD34+ cells after targeted integration into *HBB*. For example, SFFV-GFP- and PGK-GFP-positive HSPCs display a GFP MFI of 1.7×10^6 and 2.5×10^5, respectively, whereas the percent targeting efficiencies are very similar between the two promoters (~32% HR for SFFV versus ~27% HR for PGK). We also see that PGK-GFP-positive HSPCs have a lower GFP MFI than EF1α-GFP while showing twice the targeting efficiency (~27% HR for PGK versus ~14% HR for EF1α). These differences are possibly due to CD34+ cells being a very heterogeneous population of cells, with each promoter active and inactive in different populations of cells and when a promoter is active, showing a different strength of expression when activated. Of the human promoters tested, both UBC and PGK gave rise to frequencies of GFP^+ cells comparable to the non-clinically relevant viral SFFV promoter (because of both its potential enhancer activity to activate neighboring genes and it being more susceptible to silencing). This identifies them as prime candidates for enrichment and expansion of gene-modified HSPCs for use in both a research and a clinical setting. The smaller size of the PGK promoter may, in certain circumstances, be important because of the limited packaging size of AAV6. Whereas we have only compared the effect of these promoters at a single locus, this work serves as a platform for future work to drive the expression of a targeted transgene using clinically relevant promoters at other loci in HPSCs.

It has been shown that culturing human-cord-blood-derived HSPCs in low-density cell culture stimulates self-renewal of repopulating LT-HSCs.[44,45] Because HR has been suggested to be intimately linked with cell cycling, we hypothesized that, by promoting HSPCs to cycle prior to gene targeting, we would be able to prime them for HR. Though plating cells at low density in the presence of UM171/SR1 yielded no difference in frequency of DSBs or rAAV6 transduction, we show a positive correlation between the level of expansion before targeting and HR (Figure 4C). Importantly, we were able to demonstrate that this observation is $CD34^+$ cell source (CB and PB) and donor (genetics) independent, albeit with some donors showing more of a response than others. Furthermore, the increase in editing rates *in vitro* by culturing cells at low density translates to higher frequencies of engrafted gene-edited cells *in vivo*, without any loss in the level of human chimerism in mice. Whereas we hypothesize that culturing cells at low density results in more cells engaged in the G_2/M or S phases of the cell cycle,[40] which in turn primes LT-HSCs for HR, future mechanistic studies will have to address these questions. Nonetheless, these observations have large implications for increasing the absolute number of gene-targeted cells for CRISPR/Cas9 genome editing of HSPCs for translational and basic research purposes.

We demonstrate that low cell density culture conditions, using a UM171/SR1 drug combination, can expand gene-edited LT-HSCs *ex vivo*. We show that the gene-edited HSPC populations, and more importantly the rare gene-edited LT-HSCs, expand over the course of twelve days post-targeting in the presence of cytokines and these two small molecules. It has previously been reported by our group and others that LT-HSCs undergo gene targeting at a lower frequency compared to short-lived progenitors.[5,6,11] Given

the limited availability of gene-edited HSPCs in our expansion study, we used intra-bone injection of HSCs to robustly compare engraftment of cells across a range of cell concentrations and culture conditions. The use of intra-bone transplantation of HSPCs has been demonstrated to provide significantly better frequencies of engraftment of human HSPCs into immunodeficient mice when the total number of LT-HSCs is limited. This increase in human cell engraftment may be the result of decreased loss of LT-HSCs when transiting through peripheral organs, such as the lung, liver, and spleen. There is also a concomitant reduction in the kinetics of cells delivered intrabone to mobilize to other parts of the body, which we have also observed here.[53] Whereas others have previously shown that unmodified HSPCs and HSPCs with lentiviral vector integrations are able to expand *ex vivo*,[44–46] here, we provide the first demonstration that Cas9/sgRNA genome-edited HSPCs can be expanded at low-density conditions. Because it required transplantation of more *HBB*-targeted HSPCs to result in bi-lineage engraftment between days 4 and 12 post-targeting (Figure 6E; Table 1), future studies will investigate the LT-HSC cell frequency in the limiting dilution setting during expansion in order to achieve bi-lineage engraftment in all of the mice transplanted. Nevertheless, this protocol can be particularly useful to expand cells edited at a locus that undergo HR at low frequencies. The protocol could also be used to expand an enriched (>90%) edited LT-HSC population in order to yield a large enough cell population that supports durable engraftment in a patient. For diseases that require relatively high levels of engraftment of corrected cells in order to provide a significant clinical benefit, this approach might be of great utility.

In conclusion, we provide an optimized protocol for the manufacturing of gene-targeted autologous HSPCs for the treatment of the β-hemoglobinopathies. Whereas our study has focused on editing LT-HSCs at one specific locus, our work serves as a foundation for editing HSPCs at all other loci in the interest of both basic research and the treatment of hematological disease.

MATERIALS AND METHODS

AAV Vector Design and AAV6 Production and Purification

All single-stranded AAV vectors were cloned into pAAV-MCS plasmid (Agilent Technologies) using Gibson Assembly Mastermix (New England Biolabs). The *HBB* rAAV6 donors contained arms of homology to the β-globin locus of 540 bp on the left side and 420 bp on the right side (Figure 2A). Each donor also contained MaxGFP, BGH polyA, and a promoter. Self-complementary rAAV6 was made by replacing the CAG promoter in sc-AAV-CAG-GFP plasmid (kindly provided by M. Kay) with the SFFV promoter (Figure S2). rAAV6 vectors were produced as described previously.[5,54] Briefly, 293T cells seeded the previous day at 1.1×10^7 cells per 15-cm dish were transfected using polyethylenimine (PEI) with 6 μg of pAAV-MCS plasmid containing the donor along with 22 μg of pDGM6 (kindly provided by D. Russell). Cells were then lysed by three freeze-thaw cycles and treated with Benzonase, and rAAV6 particles were purified by iodixanol density gradient centrifugation. Extracted rAAV6 was then exchanged in PBS with 5% sorbitol using either a 1×10^4 molecular weight cut off (MWCO) Slide-A-Lyzer G2 dialysis Cassette (Thermo Fisher Scientific) or an Amicon centrifugal filter 1×10^5 MWCO (Millipore Sigma) following the manufacturer's instructions. Titers were measured after buffer exchange as described previously.[55]

Cell Culture

$CD34^+$ HSPCs derived from mobilized peripheral blood were purchased from AllCells, and $CD34^+$ HSPCs derived from cord blood were provided from donors under informed consent via the Binns Program for Cord Blood Research at Stanford University. $CD34^+$ donors were either used fresh or frozen immediately after isolation for later use. All $CD34^+$ HSPCs were cultured in StemSpan SFEM II (StemCell Technologies), supplemented with TPO (100 ng/mL), SCF (100 ng/mL), Flt 3 ligand (100 ng/mL), interleukin-6 (IL-6) (100 ng/mL), Stem Regenin 1 (0.75 μM; Cellagen Tech), UM171 (35 nM; StemCell Technologies), streptomycin (20 U/mL), and penicillin (20 U/mL). K562 cells were cultured in RPMI 1640 (HyClone) supplemented with 10% bovine growth serum, 100 U/mL streptomycin, 100 units/mL penicillin, and 2 mM L-glutamine. All cells were cultured at 37°C with 5% CO_2, K562 cells were cultured at ambient O_2, and $CD34^+$ HSPCs were cultured at 5% O_2. Cells were cultured at densities ranging from 1×10^5–1×10^6 cells/mL.

Production and Isolation of HF1 Cas9 Protein

A pET21 plasmid harboring the *S. pyogenes* cas9 gene was used as a template to generate HiFi cas9 (also known as HF1)[23] mutant (N496A, R661A, Q695A, and Q926A) using the GeneArt Site-Directed Mutagenesis System according to the manufacturer's protocol (Thermo Fisher Scientific). HiFi Cas9 was expressed in *Escherichia coli* and isolated as described previously.[56]

Electroporation and Targeting of Cells

The *HBB* R-02 guide was purchased from TriLink BioTechnologies and high-performance liquid chromatography (HPLC) purified. The modified sgRNA had 2′-O-methyl-3′-phosphorothioate modifications at the three terminal nucleotides of the 5′ and 3′ ends. The sequence for the R-02 guide is as follows: 5′-CTTGCCCCACAGGG CAGTAACGG-3′.[21] Cas9 protein was purchased from Thermo Fisher Scientific and Integrative DNA Technologies (IDT), and RNP was made by complexing with sgRNA at a molar ratio of 1:2.5 (Cas9:sgRNA) at 25°C for 10 min prior to electroporation. Both K562 and $CD34^+$ cells were electroporated with Cas9 at a concentration of 150–300 μg/mL. K562 cells were electroporated using the Lonza 2B Nucleofector (program T-016) in nucleofection buffer containing 100 mM KH_2PO_4, 15 mM $NaHCO_3$, 12 mM $MgCl_2 \times 6H_2O$, 8 mM ATP, and 2 mM glucose. $CD34^+$ cells were resuspended in either P3 buffer (Lonza) or buffer 1M[57] and electroporated using either the Lonza 2B (Program U-14) or Lonza 4D Nucleofector (program DZ100). Cells were plated at 5×10^5–1×10^6 cells/mL following electroporation and split to 1×10^5–2.5×10^5 cells/mL 12–24 hr following electroporation. rAAV6 was delivered onto cells immediately upon plating after electroporation at an MOI of 5×10^4–1×10^5. GFP mRNA was purchased through Trilink, GFP protein from

MilliporeSigma, and CF dye (CF488A) labeled dextran 1 × 10^4 molecular weight (MW) from Biotum. Lentivirus was purchased from Dharmacon (SPP1; GE). For experiments in which endocytosis was inhibited cells were first placed at 4°C for one hour prior to electroporation or the addition of AAV6 or dextran. After 30 min of exposure, cells were then washed two times in PBS before being returned to culture at 37°C.

Flow Cytometry and Fluorescence-Activated Cell Sorting

The percentage of cells that had undergone HR was measured by transgene expression using flow cytometry as described previously.[5] Cells were analyzed for viability and GFP expression using either the Accuri C6 flow cytometer (BD Biosciences) or a FACS Aria II SORP (BD Biosciences). Cell death was measured using annexin V (Invitrogen) and propidium iodide (BioLegend) or with propidium iodide alone following the manufacturer's instructions. Cells were stained for CD34 using anti-CD34 antibody conjugated to APC-Cy7 (clone 561; BioLegend).

Measuring Indel Frequencies

Frequencies of indels in cells were analyzed using TIDE software with sequenced PCR products of genomic DNA, extracted 4 days after electroporation as previously described.[58] Primers used for amplifying PCR fragments for TIDE at the β-globin locus are as follows: *HBB* TIDE primer forward (Fw) 5′-CCAACTCCTAAGCCAGTGC CAGAAGAG-3′ and *HBB* TIDE primer reverse (Rv) 5′-AGTCAG TGCCTATCAGAAACCCAAGAG-3′. Primers used to amplify PCR fragments for the known off-target site of guide R-02 are as follows: off-target TIDE Fw 5′-GATTGGAACCATGGGAAGCATG-3′ and off-target TIDE Rv 5′-CTCCAGTTTCTAAGAGCGGTGG-3′.

ddPCR

All guide DNA (gDNA) samples used in ddPCR were first extracted using QuickExtract DNA Extraction Solution (Epicenter) and then digested in 20 units of HINDIII-HF following the manufacturer's instructions (New England Biosciences). 3 μL of gDNA sample was then used in 25 μL ddPCR Supermix for Probes (no dUTP; Bio-Rad) following the manufacturer's instructions. Using an in-out PCR (one primer binding to integrated insert and other primer binding to *HBB* locus outside of the homology arm), a primer/probe set "*HBB* integrant" was used to quantify integrated alleles. Primer probe set "CCR2" was used to normalize to total number of alleles (Figure S4). Therefore, to generate the percentage of *HBB* alleles targeted, we divided the number of Poisson-corrected *HBB* integrant copies/μL by the number of Poisson-corrected CCR2copies/μL. Probes were at a ratio of 1:3.6 with primers. Droplet samples were prepared using 20 μL of the PCR mix and 70 μL droplet generation oil, and 40 μL of the droplet sample was used in PCR amplification. PCR cycling conditions are as follows: 98°C (10 min); 94°C (0.5 min); 60°C (0.5 min); 72°C (2 min; 50 cycles); and 98°C (10 min). Finally, droplets were analyzed according to the manufacturer's instructions using the QX200 system (Bio-Rad). The primers used to amplify integrated transgenes are as follows: *HBB* integrant primer Fw 5′-GGGAAGA CAATAGCAGGCAT-3′; *HBB* integrant primer Rv 5′-CGATCCT GAGACTTCCACAC-3′; αvδ *HBB* integrant probe 5′-FAM-TGG GGATGCGGTGGGCTCTATGGC-BHQ-3′. The primers used to amplify the CCR2 gene were as follows: CCR2 primer Fw 5′- GCTGT ATGAATCCAGGTCC-3′; CCR2 primer Rv 5′-CCTCCTGGCTGA GAAAAAG-3′; and CCR2 Probe 5′-HEX- TGTTTCCTCCAGGA TAAGGCAGCTGT-BHQ-3′.

Expansion of HSPCs

$CD34^+$ HSPCs were expanded in culture as described previously with the following modifications.[44,45] $CD34^+$ HSPCs upon thawing or purification were plated at 1 × 10^5 cells/mL on flat-bottom dishes unless stated otherwise in the figure legends. After two days, cells were targeted as described above and then transduced with rAAV6 at 1 × 10^6 cells/mL to promote transduction. After an overnight incubation, cells were diluted back down to 1 × 10^5 cells/mL. Cells were then counted and diluted down to 1 × 10^5 cells/mL once every two days.

Immunophenotyping *HBB*-Targeted HSPCs for LT-HSC Cell Surface Markers

HBB-targeted HSPCs were stained for hematopoietic stem and progenitor cell surface markers at days 4 or 12 post-targeting. Cells were spun down at 300 × *g* for 5 min and resuspended in 100 μL staining buffer containing an antibody mastermix supplied with the following human specific antibodies: 2 μL CD34-APC-Cy7 (clone 561; BioLegend); 2 μL CD45RA-BV605 (clone HI100; BioLegend); 2.5 μL CD90-BV421 (clone 5E10; BD Biosciences); and 5 μL CD201 (EPCR)-APC (BioLegend). Cells were incubated on ice for 30 min in the dark, washed with PBS, and resuspended in FACS buffer containing 1 μg/mL propidium iodide and analyzed on a FACS Aria II SORP.

Transplantation of $CD34^+$ HSPCs into NSG Mice

Six- to eight-week-old NSG mice (Jackson Laboratory, Bar Harbor) were used for *in vivo* studies. The experimental protocol was approved by Stanford University's Administrative Panel on Laboratory Animal Care. Mice were transplanted with human $CD34^+$ HSPCs 12–24 hr after 200 rads of irradiation. For tail vein injections, 350,000 *HBB*-targeted HSPCs were injected using an insulin syringe with a 27G, 0.5-inch (12.7 mm) needle. For intrafemorally injections, a 27G needle was used to create a passage through patella of mice and a 2G needle was then used to transplant cells through the patella and into the femur. The number of cells transplanted into mice varied depending on the experiment. For Figure 1E, 5 × 10^4 cord-blood-derived *HBB*-edited HSPCs were transplanted for primary transplants and the mononuclear cells (MNCs) were harvested from the bone marrow and 4.4 × 10^6 MNCs were transplanted into 2 female NSG mice for secondary engraftments to analyze repopulating potential. For Figure 5B, 3.5 × 10^5 cord-blood-derived *HBB*-edited HPSCs were transplanted intravenously for primary transplants 2 days post-targeting, 4 NSG mice receiving cells cultured in a culture density of 1 × 10^6 cells/mL, and 6 NSG mice receiving cells cultured in a culture density of 1 × 10^5 cells/mL (n = 2 CD34+ CB donors). Femurs were flushed to collect MNCs, red blood cells were lysed, and then cells were stained and analyzed for repopulating potential. For

Figures 6C and 6D, 1×10^5, 3×10^5, or 1×10^6 $CD34^+/GFP^{high}$ HSPCs were transplanted into NSG mice (at least 3 mice per group per cell dose) at day 4 or 12 post-targeting. For Figure 6E, 5×10^4 $CD34^+/GFP^{high}$ HSPCs were transplanted into ten NSG mice at day 4 post-targeting. Meanwhile, the same 5×10^4 $CD34^+/GFP^{high}$ HSPCs were cultured for an additional 8 days and then total cells were transplanted into nine NSG mouse (>2×10^6 total cells). For Figure 6F, the MNCs of primary mice were harvested after 16 weeks from only the right femur and transplanted intrafemorally into 4 NSG for secondary engraftments to analyze repopulating potential. Analysis of the 4 NSG secondary mice was carried out (16 weeks post-primary transplant harvest) by performing right femur flushes and assessing engraftment and repopulating potential of those cells.

Assessment of Human Engraftment

Sixteen weeks after transplantation, mice were euthanized and mouse bones were collected and processed as follows. For Figure 1E, 2× femur, 2× tibia, 2× humerus, sternum, 2× pelvis, and spine were collected and crushed using a mortar and pestle. Mononuclear cells were enriched using Ficoll gradient centrifugation (Ficoll-Paque Plus, GE Healthcare) for 25 min at 2,000 × *g* at room temperature. For samples analyzed at week 8 after transplant, bone marrow aspirates were taken from the right femur for analysis, and for all other studies, femurs were taken from mice and flushed with a 25G needle. Cells were then treated with red blood cell lysis buffer (1× RBC buffer; Invitrogen) for ten minutes. All samples were then blocked (10% v/v; TruStain FcX, BioLegend) and stained for 30 min at 4°C with the following antibodies before analysis: monoclonal anti-human CD45 V450 (HI30; BD Biosciences); HLA-ABC APC-Cy7 (W6/32; BioLegend); CD19 APC (HIB19; BD Biosciences); anti-mouse PE-Cy5 mTer 119 (TER-119; eBioscience); and anti-mouse CD45.1 PE-Cy7 (A20; eBioScience). Multi-lineage engraftment was defined as the presence of myeloid cells (CD33+) and B cells (CD19) within engrafted human cells (CD45+; HLA/ABC+ cells).

Statistics

All statistical analyses were performed using Prism 7 (GraphPad Software). The specifics of each statistical analysis are noted in the figure legends.

SUPPLEMENTAL INFORMATION

Supplemental Information includes seven figures and can be found with this article online at https://doi.org/10.1016/j.omtn.2018.04.017.

AUTHOR CONTRIBUTIONS

C.T.C. and J.C. contributed equally to this work as well as performed and designed most of the experiments presented here. M.K.C. and S.V. performed experiments related to Figure 2. R.O.B. performed experiments related to Figure 6. J.M.C. and J.P. produced the HF1 Cas9. D.P.D. performed experiments throughout the study. C.T.C. and J.C. wrote the manuscript with help from all of the authors. D.P.D. and M.H.P. equally directed the research and participated in the design and interpretation of the experiments and the writing of the manuscript.

CONFLICTS OF INTEREST

M.H.P. is a consultant and has equity interest in CRISPR Tx, but CRISPR Tx had no input or opinions on the subject matter relayed herein. J.P. and J.M.C. are employees of Thermo Fisher Scientific.

ACKNOWLEDGMENTS

We would like to thank D. Russell for the pDGM6 plasmid, Hans-Peter Kiem for scAAV6, Jan Carrette for AAVR KO cells, and the Binns Program for Cord Blood Research at Stanford University for cord-blood-derived $CD34^+$ HSPCs. We also would like to give thanks to the members of the Porteus laboratory for input, comments, and discussion. M.H.P. gratefully acknowledges the support of the Amon Carter Foundation, the Laurie Kraus Lacob Faculty Scholar Award in Pediatric Translational Research, the Sutardja Foundation, and NIH grant support (R01-AI097320 and R01-AI120766). D.P.D. was supported by the Stanford Child Health Research Institute (CHRI) grant and the Bass Cancer Center Program Endowment. R.O.B. was supported through an Individual Postdoctoral grant (DFF–1333-00106B) and a Sapere Aude, Research Talent grant (DFF–1331-00735B), both from the Danish Council for Independent Research, Medical Sciences. C.T.C. was supported by a CIRM Bridges grant (CIRM TB1-01190).

REFERENCES

1. Lucarelli, G., Gaziev, J., Isgrò, A., Sodani, P., Paciaroni, K., Alfieri, C., De Angelis, G., Marziali, M., Simone, M.D., Gallucci, C., et al. (2012). Allogeneic cellular gene therapy in hemoglobinopathies–evaluation of hematopoietic SCT in sickle cell anemia. Bone Marrow Transplant. *47*, 227–230.

2. Voit, R.A., Hendel, A., Pruett-Miller, S.M., and Porteus, M.H. (2014). Nuclease-mediated gene editing by homologous recombination of the human globin locus. Nucleic Acids Res. *42*, 1365–1378.

3. Hamosh, A., and Stumpf, A.M. (2013). Sickle cell anemia. In Online Mendelian Inheritance in Man, OMIM (Baltimore, MD: John Hopkins University).

4. Ribeil, J.-A., Hacein-Bey-Abina, S., Payen, E., Magnani, A., Semeraro, M., Magrin, E., Caccavelli, L., Neven, B., Bourget, P., El Nemer, W., et al. (2017). Gene therapy in a patient with sickle cell disease. N. Engl. J. Med. *376*, 848–855.

5. Dever, D.P., Bak, R.O., Reinisch, A., Camarena, J., Washington, G., Nicolas, C.E., Pavel-Dinu, M., Saxena, N., Wilkens, A.B., Mantri, S., et al. (2016). CRISPR/Cas9 β-globin gene targeting in human haematopoietic stem cells. Nature *539*, 384–389.

6. DeWitt, M.A., Magis, W., Bray, N.L., Wang, T., Berman, J.R., Urbinati, F., Heo, S.J., Mitros, T., Muñoz, D.P., Boffelli, D., et al. (2016). Selection-free genome editing of the sickle mutation in human adult hematopoietic stem/progenitor cells. Sci. Transl. Med. *8*, 360ra134.

7. Barrangou, R., Fremaux, C., Deveau, H., Richards, M., Boyaval, P., Moineau, S., Romero, D.A., and Horvath, P. (2007). CRISPR provides acquired resistance against viruses in prokaryotes. Science *315*, 1709–1712.

8. Porteus, M.H. (2015). Towards a new era in medicine: therapeutic genome editing. Genome Biol. *16*, 286.

9. Hoban, M.D., Cost, G.J., Mendel, M.C., Romero, Z., Kaufman, M.L., Joglekar, A.V., Ho, M., Lumaquin, D., Gray, D., Lill, G.R., et al. (2015). Correction of the sickle cell disease mutation in human hematopoietic stem/progenitor cells. Blood *125*, 2597–2604.

10. Hendel, A., Bak, R.O., Clark, J.T., Kennedy, A.B., Ryan, D.E., Roy, S., Steinfeld, I., Lunstad, B.D., Kaiser, R.J., Wilkens, A.B., et al. (2015). Chemically modified guide RNAs enhance CRISPR-Cas genome editing in human primary cells. Nat. Biotechnol. *33*, 985–989.

11. Genovese, P., Schiroli, G., Escobar, G., Tomaso, T.D., Firrito, C., Calabria, A., Moi, D., Mazzieri, R., Bonini, C., Holmes, M.C., et al. (2014). Targeted genome editing in human repopulating haematopoietic stem cells. Nature *510*, 235–240.

12. Storici, F., Snipe, J.R., Chan, G.K., Gordenin, D.A., and Resnick, M.A. (2006). Conservative repair of a chromosomal double-strand break by single-strand DNA through two steps of annealing. Mol. Cell. Biol. *26*, 7645–7657.

13. Wang, J., Exline, C.M., DeClercq, J.J., Llewellyn, G.N., Hayward, S.B., Li, P.W.-L., Shivak, D.A., Surosky, R.T., Gregory, P.D., Holmes, M.C., and Cannon, P.M. (2015). Homology-driven genome editing in hematopoietic stem and progenitor cells using ZFN mRNA and AAV6 donors. Nat. Biotechnol. *33*, 1256–1263.

14. De Ravin, S.S., Reik, A., Liu, P.-Q., Li, L., Wu, X., Su, L., Raley, C., Theobald, N., Choi, U., Song, A.H., et al. (2016). Targeted gene addition in human CD34(+) hematopoietic cells for correction of X-linked chronic granulomatous disease. Nat. Biotechnol. *34*, 424–429.

15. Sather, B.D., Romano Ibarra, G.S., Sommer, K., Curinga, G., Hale, M., Khan, I.F., Singh, S., Song, Y., Gwiazda, K., Sahni, J., et al. (2015). Efficient modification of CCR5 in primary human hematopoietic cells using a megaTAL nuclease and AAV donor template. Sci. Transl. Med. *7*, 307ra156.

16. Bak, R.O., Dever, D.P., and Porteus, M.H. (2018). CRISPR/Cas9 genome editing in human hematopoietic stem cells. Nat. Protoc. *13*, 358–376.

17. Bak, R.O., Dever, D.P., Reinisch, A., Cruz Hernandez, D., Majeti, R., and Porteus, M.H. (2017). Multiplexed genetic engineering of human hematopoietic stem and progenitor cells using CRISPR/Cas9 and AAV6. eLife *6*, e27873.

18. Bak, R.O., and Porteus, M.H. (2017). CRISPR-mediated integration of large gene cassettes using AAV donor vectors. Cell Rep. *20*, 750–756.

19. Gundry, M.C., Brunetti, L., Lin, A., Mayle, A.E., Kitano, A., Wagner, D., Hsu, J.I., Hoegenauer, K.A., Rooney, C.M., Goodell, M.A., and Nakada, D. (2016). Highly efficient genome editing of murine and human hematopoietic progenitor cells by CRISPR/Cas9. Cell Rep. *17*, 1453–1461.

20. Hultquist, J.F., Schumann, K., Woo, J.M., Manganaro, L., McGregor, M.J., Doudna, J., Simon, V., Krogan, N.J., and Marson, A. (2016). A Cas9 ribonucleoprotein platform for functional genetic studies of HIV-host interactions in primary human T cells. Cell Rep. *17*, 1438–1452.

21. Cradick, T.J., Fine, E.J., Antico, C.J., and Bao, G. (2013). CRISPR/Cas9 systems targeting β-globin and CCR5 genes have substantial off-target activity. Nucleic Acids Res. *41*, 9584–9592.

22. Doyon, Y., Choi, V.M., Xia, D.F., Vo, T.D., Gregory, P.D., and Holmes, M.C. (2010). Transient cold shock enhances zinc-finger nuclease-mediated gene disruption. Nat. Methods *7*, 459–460.

23. Kleinstiver, B.P., Pattanayak, V., Prew, M.S., Tsai, S.Q., Nguyen, N.T., Zheng, Z., and Joung, J.K. (2016). High-fidelity CRISPR-Cas9 nucleases with no detectable genome-wide off-target effects. Nature *529*, 490–495.

24. Shultz, L.D., Ishikawa, F., and Greiner, D.L. (2007). Humanized mice in translational biomedical research. Nat. Rev. Immunol. *7*, 118–130.

25. Schiroli, G., Ferrari, S., Conway, A., Jacob, A., Capo, V., Albano, L., Plati, T., Castiello, M.C., Sanvito, F., Gennery, A.R., et al. (2017). Preclinical modeling highlights the therapeutic potential of hematopoietic stem cell gene editing for correction of SCID-X1. Sci. Transl. Med. *9*, eaan0820.

26. McCarty, D.M., Monahan, P.E., and Samulski, R.J. (2001). Self-complementary recombinant adeno-associated virus (scAAV) vectors promote efficient transduction independently of DNA synthesis. Gene Ther. *8*, 1248–1254.

27. Seisenberger, G., Ried, M.U., Endress, T., Büning, H., Hallek, M., and Bräuchle, C. (2001). Real-time single-molecule imaging of the infection pathway of an adeno-associated virus. Science *294*, 1929–1932.

28. Bartlett, J.S., Wilcher, R., and Samulski, R.J. (2000). Infectious entry pathway of adeno-associated virus and adeno-associated virus vectors. J. Virol. *74*, 2777–2785.

29. Finkelshtein, D., Werman, A., Novick, D., Barak, S., and Rubinstein, M. (2013). LDL receptor and its family members serve as the cellular receptors for vesicular stomatitis virus. Proc. Natl. Acad. Sci. USA *110*, 7306–7311.

30. Amirache, F., Lévy, C., Costa, C., Mangeot, P.-E., Torbett, B.E., Wang, C.X., Nègre, D., Cosset, F.L., and Verhoeyen, E. (2014). Mystery solved: VSV-G-LVs do not allow efficient gene transfer into unstimulated T cells, B cells, and HSCs because they lack the LDL receptor. Blood *123*, 1422–1424.

31. Saftig, P., and Klumperman, J. (2009). Lysosome biogenesis and lysosomal membrane proteins: trafficking meets function. Nat. Rev. Mol. Cell Biol. *10*, 623–635.

32. Sherwood, M.W., Prior, I.A., Voronina, S.G., Barrow, S.L., Woodsmith, J.D., Gerasimenko, O.V., Petersen, O.H., and Tepikin, A.V. (2007). Activation of trypsinogen in large endocytic vacuoles of pancreatic acinar cells. Proc. Natl. Acad. Sci. USA *104*, 5674–5679.

33. Pillay, S., Meyer, N.L., Puschnik, A.S., Davulcu, O., Diep, J., Ishikawa, Y., Jae, L.T., Wosen, J.E., Nagamine, C.M., Chapman, M.S., and Carette, J.E. (2016). An essential receptor for adeno-associated virus infection. Nature *530*, 108–112.

34. Dausend, J., Musyanovych, A., Dass, M., Walther, P., Schrezenmeier, H., Landfester, K., and Mailänder, V. (2008). Uptake mechanism of oppositely charged fluorescent nanoparticles in HeLa cells. Macromol. Biosci. *8*, 1135–1143.

35. Cartiera, M.S., Johnson, K.M., Rajendran, V., Caplan, M.J., and Saltzman, W.M. (2009). The uptake and intracellular fate of PLGA nanoparticles in epithelial cells. Biomaterials *30*, 2790–2798.

36. Cao, H., Chen, J., Awoniyi, M., Henley, J.R., and McNiven, M.A. (2007). Dynamin 2 mediates fluid-phase micropinocytosis in epithelial cells. J. Cell Sci. *120*, 4167–4177.

37. Orthwein, A., Noordermeer, S.M., Wilson, M.D., Landry, S., Enchev, R.I., Sherker, A., Munro, M., Pinder, J., Salsman, J., Dellaire, G., et al. (2015). A mechanism for the suppression of homologous recombination in G1 cells. Nature *528*, 422–426.

38. Yun, M.H., and Hiom, K. (2009). CtIP-BRCA1 modulates the choice of DNA double-strand-break repair pathway throughout the cell cycle. Nature *459*, 460–463.

39. Chiruvella, K.K., Liang, Z., and Wilson, T.E. (2013). Repair of double-strand breaks by end joining. Cold Spring Harb. Perspect. Biol. *5*, a012757.

40. Karanam, K., Kafri, R., Loewer, A., and Lahav, G. (2012). Quantitative live cell imaging reveals a gradual shift between DNA repair mechanisms and a maximal use of HR in mid S phase. Mol. Cell *47*, 320–329.

41. Cheshier, S.H., Morrison, S.J., Liao, X., and Weissman, I.L. (1999). In vivo proliferation and cell cycle kinetics of long-term self-renewing hematopoietic stem cells. Proc. Natl. Acad. Sci. USA *96*, 3120–3125.

42. Kiel, M.J., He, S., Ashkenazi, R., Gentry, S.N., Teta, M., Kushner, J.A., Jackson, T.L., and Morrison, S.J. (2007). Haematopoietic stem cells do not asymmetrically segregate chromosomes or retain BrdU. Nature *449*, 238–242.

43. Mohrin, M., Bourke, E., Alexander, D., Warr, M.R., Barry-Holson, K., Le Beau, M.M., Morrison, C.G., and Passegué, E. (2010). Hematopoietic stem cell quiescence promotes error-prone DNA repair and mutagenesis. Cell Stem Cell *7*, 174–185.

44. Csaszar, E., Kirouac, D.C., Yu, M., Wang, W., Qiao, W., Cooke, M.P., Boitano, A.E., Ito, C., and Zandstra, P.W. (2012). Rapid expansion of human hematopoietic stem cells by automated control of inhibitory feedback signaling. Cell Stem Cell *10*, 218–229.

45. Fares, I., Chagraoui, J., Gareau, Y., Gingras, S., Ruel, R., Mayotte, N., Csaszar, E., Knapp, D.J., Miller, P., Ngom, M., et al. (2014). Cord blood expansion. Pyrimidoindole derivatives are agonists of human hematopoietic stem cell self-renewal. Science *345*, 1509–1512.

46. Zonari, E., Desantis, G., Petrillo, C., Boccalatte, F.E., Lidonnici, M.R., Kajaste-Rudnitski, A., Aiuti, A., Ferrari, G., Naldini, L., and Gentner, B. (2017). Efficient ex vivo engineering and expansion of highly purified human hematopoietic stem and progenitor cell populations for gene therapy. Stem Cell Reports *8*, 977–990.

47. Fares, I., Chagraoui, J., Lehnertz, B., MacRae, T., Mayotte, N., Tomellini, E., Aubert, L., Roux, P.P., and Sauvageau, G. (2017). EPCR expression marks UM171-expanded CD34+ cord blood stem cells. Blood *129*, 3344–3351.

48. Peterson, C.W., Wang, J., Norman, K.K., Norgaard, Z.K., Humbert, O., Tse, C.K., Yan, J.J., Trimble, R.G., Shivak, D.A., Rebar, E.J., et al. (2016). Long-term multilineage engraftment of autologous genome-edited hematopoietic stem cells in nonhuman primates. Blood *127*, 2416–2426.

49. Harmatz, P., Burton, B., Whitley, C., Lau, H.A., Muenzer, J., Prada, C.E., and Ficicioglu, C. (2018). Ascending dose study of genome editing by the zinc finger nuclease (ZFN) therapeutic SB-913 in subjects with MPS II. ClinicalTrials.gov. https://clinicaltrials.gov/ct2/show/NCT03041324.

50. Dupuy, F.P., Mouly, E., Mesel-Lemoine, M., Morel, C., Abriol, J., Cherai, M., Baillou, C., Nègre, D., Cosset, F.L., Klatzmann, D., and Lemoine, F.M. (2005). Lentiviral transduction of human hematopoietic cells by HIV-1- and SIV-based vectors containing a bicistronic cassette driven by various internal promoters. J. Gene Med. *7*, 1158–1171.

51. Ramezani, A., Hawley, T.S., and Hawley, R.G. (2000). Lentiviral vectors for enhanced gene expression in human hematopoietic cells. Mol. Ther. *2*, 458–469.

52. Varma, N., Janic, B., Ali, M., Iskander, A., and Arbab, A. (2011). Lentiviral based gene transduction and promoter studies in human hematopoietic stem cells (hHSCs). J. Stem Cells Regen. Med. *7*, 41–53.

53. Futrega, K., Lott, W.B., and Doran, M.R. (2016). Direct bone marrow HSC transplantation enhances local engraftment at the expense of systemic engraftment in NSG mice. Sci. Rep. *6*, 23886.

54. Khan, I.F., Hirata, R.K., and Russell, D.W. (2011). AAV-mediated gene targeting methods for human cells. Nat. Protoc. *6*, 482–501.

55. Aurnhammer, C., Haase, M., Muether, N., Hausl, M., Rauschhuber, C., Huber, I., Nitschko, H., Busch, U., Sing, A., Ehrhardt, A., and Baiker, A. (2012). Universal real-time PCR for the detection and quantification of adeno-associated virus serotype 2-derived inverted terminal repeat sequences. Hum. Gene Ther. Methods *23*, 18–28.

56. Liang, X., Potter, J., Kumar, S., Zou, Y., Quintanilla, R., Sridharan, M., Carte, J., Chen, W., Roark, N., Ranganathan, S., et al. (2015). Rapid and highly efficient mammalian cell engineering via Cas9 protein transfection. J. Biotechnol. *208*, 44–53.

57. Chicaybam, L., Sodre, A.L., Curzio, B.A., and Bonamino, M.H. (2013). An efficient low cost method for gene transfer to T lymphocytes. PLoS ONE *8*, e60298.

58. Brinkman, E.K., Chen, T., Amendola, M., and van Steensel, B. (2014). Easy quantitative assessment of genome editing by sequence trace decomposition. Nucleic Acids Res. *42*, e168.

OMTN, Volume *12*

Supplemental Information

Priming Human Repopulating Hematopoietic Stem and Progenitor Cells for Cas9/sgRNA Gene Targeting

Carsten T. Charlesworth, Joab Camarena, M. Kyle Cromer, Sriram Vaidyanathan, Rasmus O. Bak, Jason M. Carte, Jason Potter, Daniel P. Dever, and Matthew H. Porteus

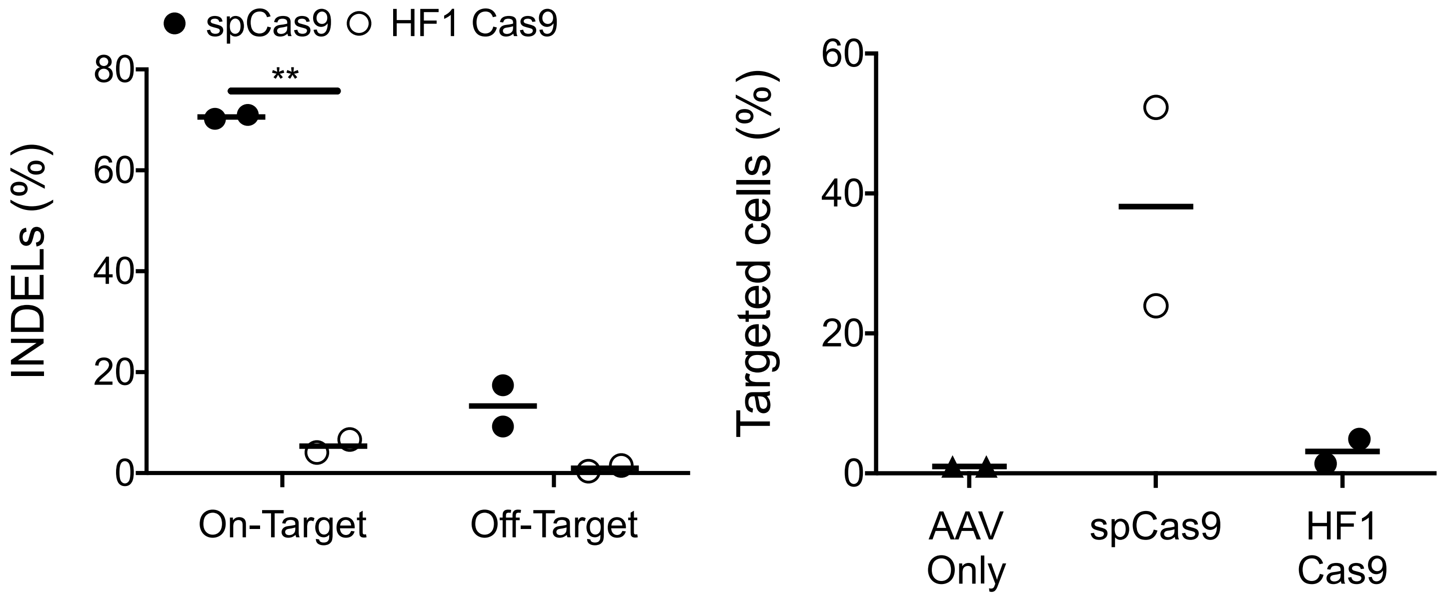

Supplemental Figure 1. CB CD34+ HSPCs were edited at the *HBB* locus using either sgRNA/Cas9 only (left) or sgRNA/Cas9 plus AAV6 (right). Left, percentage of alleles that contain INDELs at the *HBB* locus and at a known off-target site for the R-02 guide when HF1 Cas9 was used to compare to the wild type spCas9 (n = number of data points). **$P < 0.05$, paired t-test. Right, AAV6-SFFV-GFP was used to deliver a homologous donor containing GFP controlled by the SFFV promoter to cells. The percentage of cells edited was measured by GFP^{high} 4 days after targeting (n = number of data points plotted). Bars represent mean.

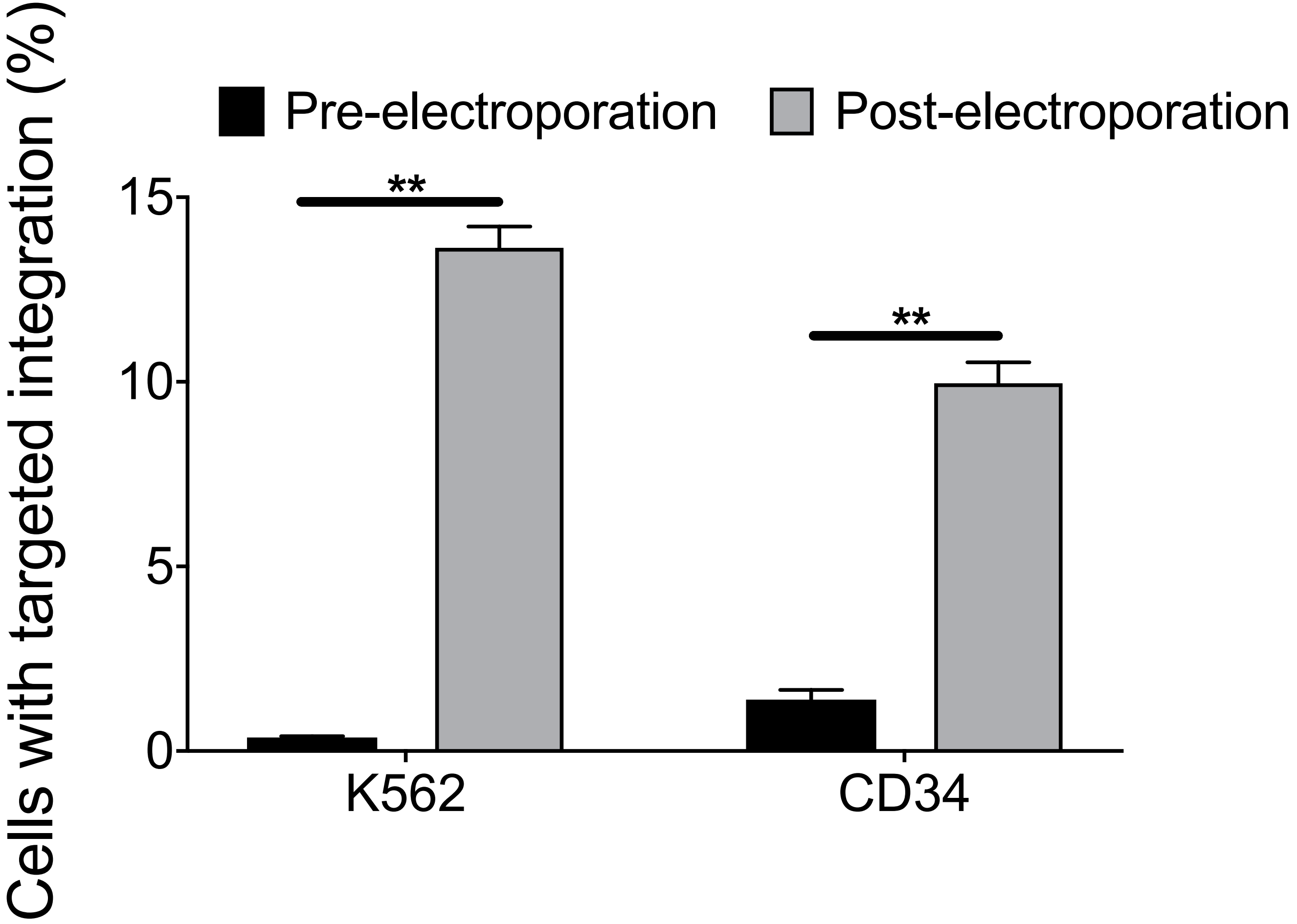

Supplemental Figure 2. Percentage of PB cells that undergo HR at the *HBB* locus when rAAV6 is added 24 hours before or directly after electroporation of cells with Cas9/sgRNA RNP at a multiplicity of infection (MOI) of 5×10^4 vg/cell (n = 3 replicates of K562 and n = 4-6 PB $CD34^+$ donors). $^{**}P < .01$, paired t-test.

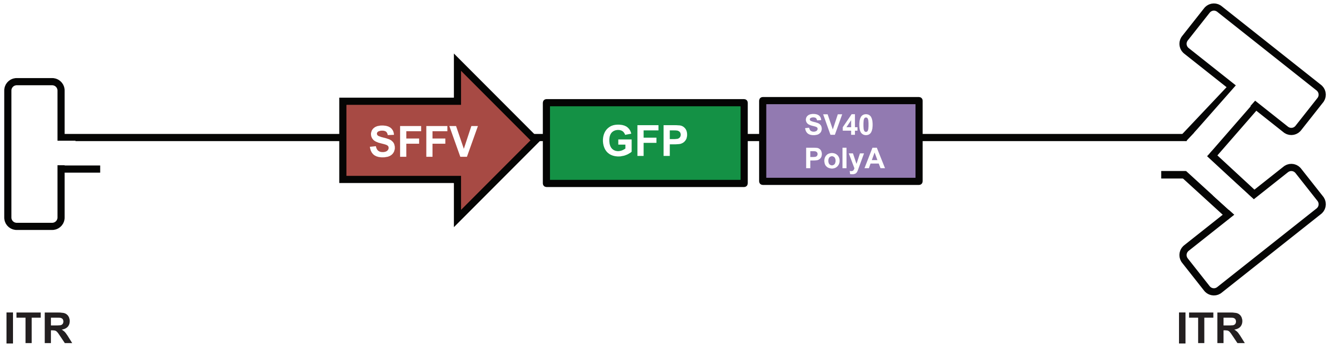

Supplemental Figure 3. Schematic showing the structure of scAAV6. ITR stands for Iinverted terminal repeat.

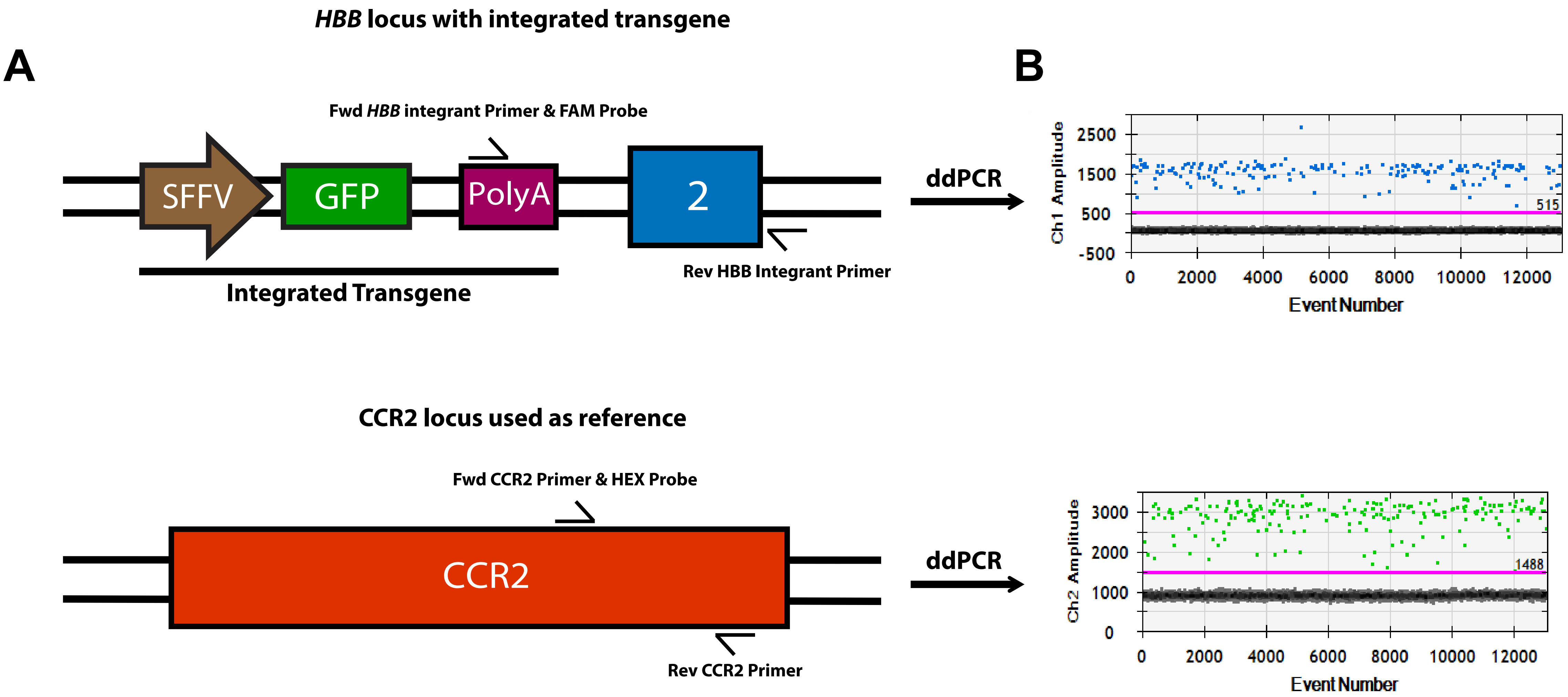

Supplemental Figure 4. Schematic demonstrating the position of probes for detecting integrated transgene donor relative to a reference gene and detection by droplet reader. **(A)** Position of primers and probes for amplifying an allele with an integrated transgene at the HBB locus using an in-out PCR (one primer binding to integrated insert with FAM probe 3 bp upstream and other primer biinding to *HBB* locus outside of the homology arm) and primers for amplifying the CCR2 locus to serve as a reference for the number of alleles edited. **(B)** Representative ddPCR readout demonstrating droplets that are positive for a transgene as detected by FAM fluorescence compared to areporter gene as detected by HEX fluorescence.

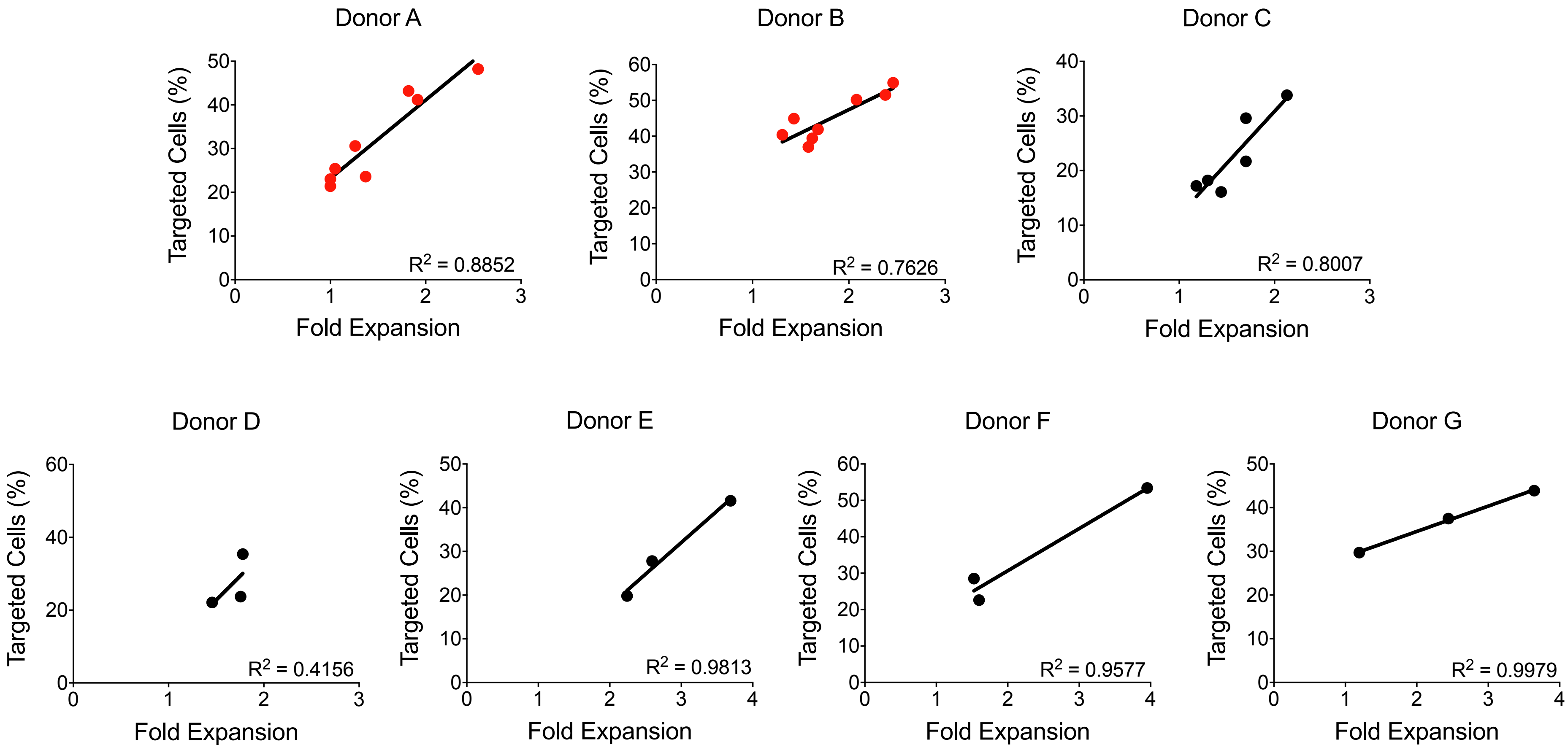

Supplemental Figure 5. Individual donors were plated at different cell densities or for different amounts of times and then targeted at the *HBB* locus. The fold expansion of cells is plotted against the % of cells in the population that underwent HR and a linear regression plotted for each donor. Red dots represent a mobilized peripheral blood donor source and black dots represent cord blood donor source.

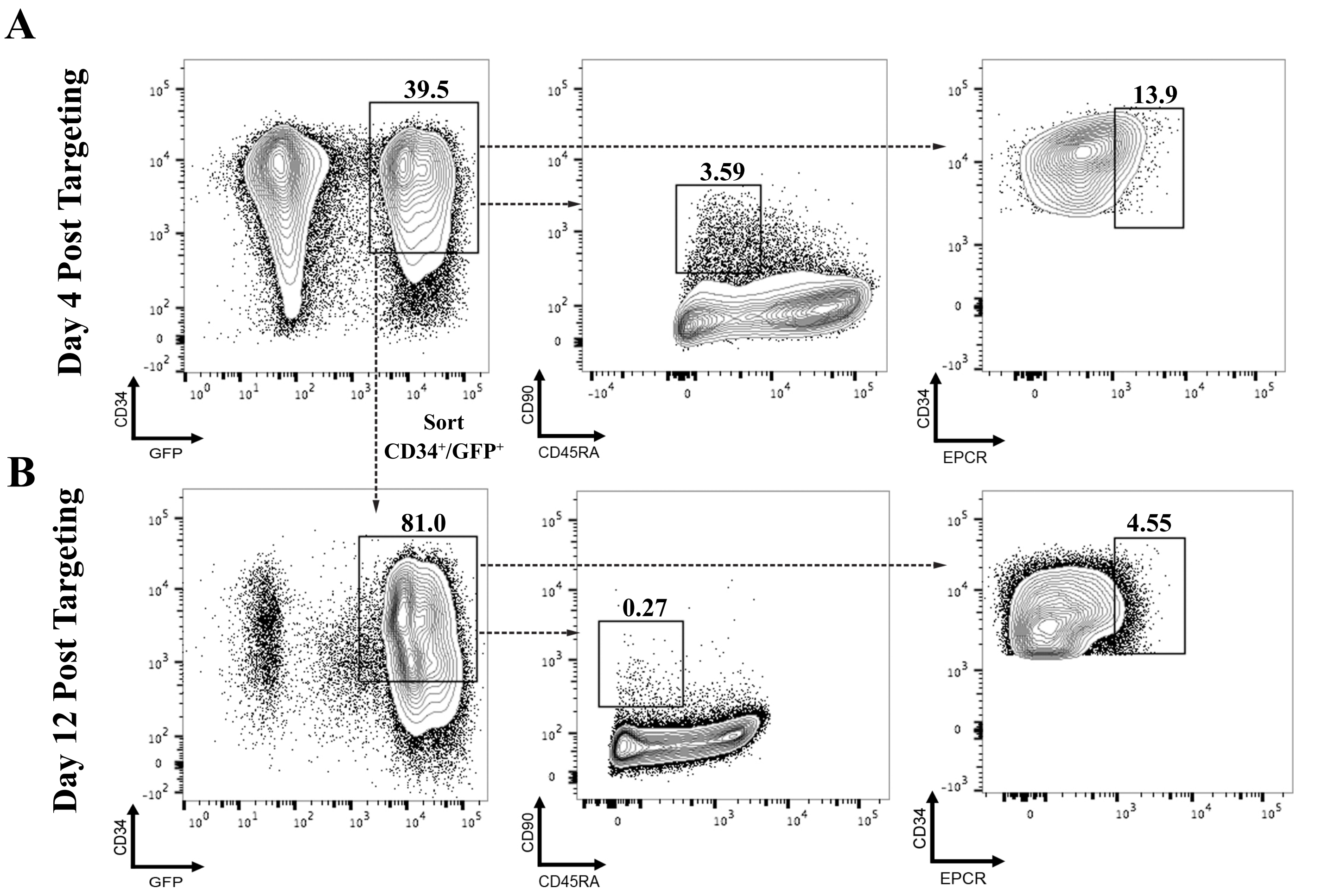

Supplemental Figure 6. Expanding CB *HBB*-targeted HSPCs for 12 days post-targeting reduces the percentage of immunophenotypic repopulating HSPCs. **A)** *HBB*-targeted HSPCs were analyzed day 4 post-targeting for $CD34^+/GFP^+$ expression (left), CD90/CD45RA expression (middle) and CD201 (EPCR) expression (right). $CD34^+/CD45RA^-/CD90^+/GFP^+$ cells indicate targeting efficiencies in repopulating HSCs. EPCR high expression (right) is indicative of HSPCs with long-term population when cultured. **B)** FACS plot showing the percentage of immunophenotypic repopulating HSPCs in *HBB*-targeted cells after 8 days of expansion at low densities. Data show the presence but a reduction in the percentage of HSPCs expressing CD90 (middle) and EPCR (right).

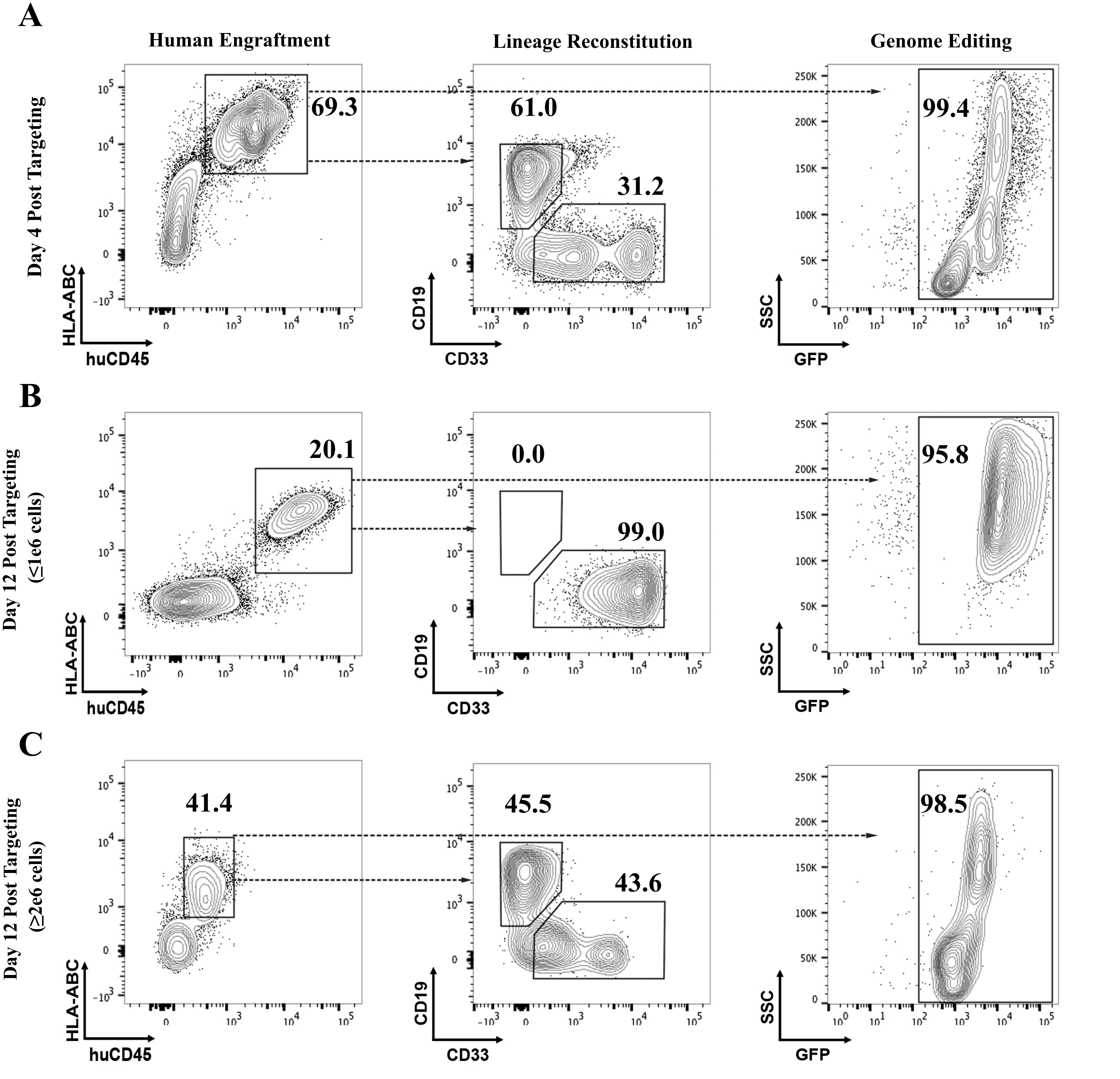

Supplemental Figure 7. Representative FACS plots from **Figure 6C-E** showing human chimerism of CB *HBB*-targeted HSPCs in NSG bone marrow. **A)** 1×10^6 $CD34^+/GFP^+$ *HBB*-targeted HSPCs day 4-post targeting were injected into the right femur of a NSG mouse. 16 weeks post transplant, the bone marrow was analyzed for human cells and further analyzed for bi-lineage reconstitution and genome editing frequencies via GFP analysis. Data shows robust human engraftment in mouse femur (left) with bi-lineage reconstitution (right) of Cas9/sgRNA modified cells (right). **B)** On day 4 post-targeting, $CD34^+/GFP^+$ HBB-targeted HSPCs were expanded for an additional 8 days and then 1×10^6 CD34+/GFP+ cells were sorted again and finally injected into the right femur of a NSG mouse. Human cells were analyzed as above. Data shows robust engraftment of human cells, but with only myeloid reconstitution. **C)** On day 4 post-targeting, 5×10^4 $CD34^+/GFP^+$ *HBB*-targeted HSPCs were sorted and expanded 57.5 fold for an additional 8 days at low densities and then injected in the right femur of a NSG mouse. Human cell chimerism, lineage reconstitution and genome editing was analyzed at 16 weeks post-targeting. Data show robust engraftment compared to Day 4 equivalent (5×10^4; **Figure 6E**) as well as multi-lineage reconstitution (middle) of a pure genome edited population (right).
